# Classical Conditioning of Adult *Drosophila*

**DOI:** 10.1101/pdb.prot108566

**Published:** 2025-08-01

**Authors:** Zeynep Okray, Pedro F. Jacob, John-Paul Moszynski, Clifford B. Talbot, Scott Waddell

**Affiliations:** Centre for Neural Circuits and Behaviour, University of Oxford, Oxford OX1 3TA, United Kingdom

## Abstract

Olfactory classical conditioning paradigms have been extensively used since the early 1970s to apply genetic approaches to the study of memory in *Drosophila*. Over the intervening years, advances in genetics have largely changed the focus of studies from the role of single genes in memory to investigation of memory-relevant neuronal circuits. However, the original behavioral paradigms have remained largely unaltered, besides investigators making a few useful tweaks to the training and testing apparatus and modifications to the operating procedures. In this protocol, we provide the reader with a detailed description of the manufacture and assembly of a typical T-maze apparatus, where populations of adult flies can be trained and their odor memory tested later, by giving them a binary choice between the two trained odors. We describe how variations of the training apparatus permit both aversive (odor–shock) and appetitive (odor–sugar) memories to be studied. In addition, we describe a recent modification of the apparatus and protocol that permits study of multisensory (color and odor) aversive and appetitive learning. Control assays for sensory acuity and locomotion are also included.

## Materials

It is essential that you consult the appropriate Material Safety Data Sheets and your institution’s Environmental Health and Safety Office for proper handling of equipment and hazardous materials used in this protocol.

RECIPES: Please see the end of this protocol for recipes indicated by <R>. Additional recipes can be found online at http://cshprotocols.cshlp.org/site/recipes.

### Reagents

Reagents for appetitive conditioning (see Steps 75–101)
  1% (w/v) agar (80–100 mesh; Fisher BioReagents BP2641-1)
    *Dissolve agar powder in tap water by microwaving until almost boiling (avoid spilling over). Pour the hot agar solution into 25-mm × 95-mm polystyrene* Drosophila *vials (Flystuff 32-109), with 2*–*2*.*5 mL per vial. Allow to set and dry, cover with cling film, and store at 4*°*C. See* Step 75.

*Drosophila* adult flies (2- to 7-d-old, ~200 per *n* required)
Mineral oil
Odors
  3-Octanol (OCT; 99%; e.g., Sigma-Aldrich 218405)
  4-Methylcyclohexanol (MCH; mixture of *cis* and *trans*; 98%; e.g., Sigma-Aldrich 153095)
  Saturated sucrose solution (5.8 M)
    *Use tap water to dissolve the sugar. We find that distilled water is less effective*.

Reagents for aversive and appetitive multisensory conditioning (see Steps 102–121)
  Lead-free solder
  Thermal paste (e.g., RS Components 271-3835)
Reagents for aversive conditioning (see Steps 41–74)
  Cornmeal fly media <R> (fly food)
  *Drosophila* adult flies (2- to 7-d-old, ~200 per *n* required)
  Mineral oil
  Odors
    3-Octanol (OCT; 99%; e.g., Sigma-Aldrich 218405)
    4-Methylcyclohexanol (MCH; mixture of *cis* and *trans*; 98%; e.g., Sigma-Aldrich 153095)
Reagents for T-maze and related equipment manufacture/assembly (see Steps 1–40)
  Ethanol (100%)
  Lead-free solder
  SUP706B support material (Objet)
  Two-component adhesive V9500 epoxy (HellermannTyton)
  VeroClear or VeroUltraClear 3D model material (Objet)

### Equipment

Equipment for appetitive conditioning (see Steps 75–101)
  3MM Whatman filter paper
  Cling film
  Fly vials (e.g., 25-mm × 95-mm polystyrene *Drosophila* vials; Flystuff 32-109)
  Foam plugs (e.g., Flystuff Flugs for 25-mm tubes; Flystuff 49-102)
    *These are used to cap fly vials*.

Equipment for aversive and appetitive multisensory conditioning (see Steps 102–121)
  Aluminium LED printed circuit boards PCBs (two)
    *CAD files are available at* https://github.com/CNCBWaddellTmaze/TMaze/releases/tag/TMaze-v1.0.
  Bag, black, opaque (one; e.g., a heavy-duty black bin bag)
  Double-sided heatsink tape (RS Components 707-4828)
  Heatsinks, two per T-maze, one for each LED color (e.g., RS Components 271-864)
  LED connection cable (e.g., Thorlabs CAB-LEDD1)
  LED controller (set to 0.7 A) (e.g., Thorlabs LEDD1B)
    *We use an equivalent, in-house-built device based on the Recom RCD-24-0*.*70/Vref LED driver*.
  LED controller power supply for LED driver (e.g., Thorlabs KPS201)
  LEDs, blue, wavelength 465-nm ± 10-nm (e.g., ProLight Opto PM2B-3LDE-SD)
    *One blue LED unit consists of four blue LEDs mounted on a heatsink. One blue LED unit is needed per T-maze*.
  LEDs, green, wavelength 530-nm ± 10-nm (e.g., ProLight Opto PM2E-3LGE-SD)
    *One green LED unit consists of four green LEDs mounted on a heatsink. One green LED unit is needed per T-maze*.
  M3 nuts (two)
  M3 screws, ~10-mm in length (two)
  PCB covers (two)
    *CAD files are available at* https://github.com/CNCBWaddellTmaze/TMaze/releases/tag/TMaze-v1.0.
  Self-adhesive plastic sheets (black, matte; e.g., Greenstar Graphics matte A4 sticky-back plastic self-adhesive vinyl)
  Soldering iron/station

Equipment for aversive conditioning (see Steps 41–74)
  3MM Whatman filter paper (Cytiva Whatman 3-mm paper 3030-917)
  Fly vials (e.g., 25-mm × 95-mm polystyrene *Drosophila* vials; Flystuff 32-109)
  Foam plugs (Flystuff Flugs for 25-mm tubes; Flystuff 49-102)
    *These are used to cap fly vials*.

Equipment for bench setup (see Steps 32–36)
  Behavior room/chamber, temperature- and humidity-controlled
    *Set the temperature to 23*°*C unless temperature-sensitive alleles/transgenes are used. For aversive training, the behavior room humidity is crucial (it should not be <55%;* ~*70% is optimal)*.
  BNC cable
  Calibrated ball flowmeter to measure airflow (e.g., RS PRO for Air Flow Sensor for Air RS 257-6415)
  Data logger for temperature and humidity
  Millipore filters (e.g., Merck Millex hydrophobic PTFE vent filter; Sigma Aldrich SLFH050) Needle valves (Festo GR Series Flow Valve 193967; RS 175-4610)
  Neoprene sheeting, two pieces of 100-cm × 50-cm (e.g., Neoprene Sponge Plain, MacLellan Rubber Limited)
  S48 stimulator (Grass Instruments, Astro-Med, Inc.)
    *Alternatively, a battery-operated stimulator can be used (e*.*g*., *a combination of a DS2A Isolated Voltage Stimulator and DG2A Train Delay Generator [Digitimer Ltd])*.
  Thick tubing to connect the vacuum port to the T-maze apparatus (e.g., rubber tubing with 17-mm OD and 8-mm ID)
  Timers
  Vacuum pump and port
Equipment for fly collection equipment assembly (see Steps 37–40)
  Cardboard box and divider (e.g., FlyStuff cardboard trays, 26.7-cm × 26.7-cm, 5.08-cm, SLS FLY1024; and dividers, SLS FLY1018)
    *These are used to store collection tubes*.
  Foam sponge
*Cut in a circle with diameter 5-cm, height 3-cm*
  Printed parts from Table 1
  Polystyrene round-bottom tubes, 14-mL (e.g., Falcon 14-mL round-bottom polypropylene test tubes with snap caps 17-mm × 100-mm; SLS 352059)
    *These are used as collection tubes*.

Equipment for odor delivery vial assembly (see Steps 25–31)
  Bunsen burner
  Diamond glass cutter
  Hardware parts from Table 2
  Parafilm
Equipment for switch box (see Steps 23–24)
  Hardware parts from Table 3
Equipment for T-maze and tubes manufacture/assembly (see Steps 1–22)
  28 thread tap (tapered), 0.25-in
  3D printer (e.g., Objet 260 Connex3 PolyJet printer)
  Electric drill with 4-mm bit
  Electrical tape
  Hardware and printed parts from Tables 2 and 4
  Heat-shrink tubing and heat gun
  Hex keys of various sizes (0.70-mm required)
  Hot glue gun and hot glue
  Label maker
  M6 thread tap (tapered, bottoming)
  Plastic bristle scrubbing brush
  Retractable knife
  Self-fusing silicone tape, 25-mm wide, transparent
  Soldering iron/station
  Wrench (adjustable)

## Method

**Figure 1:**
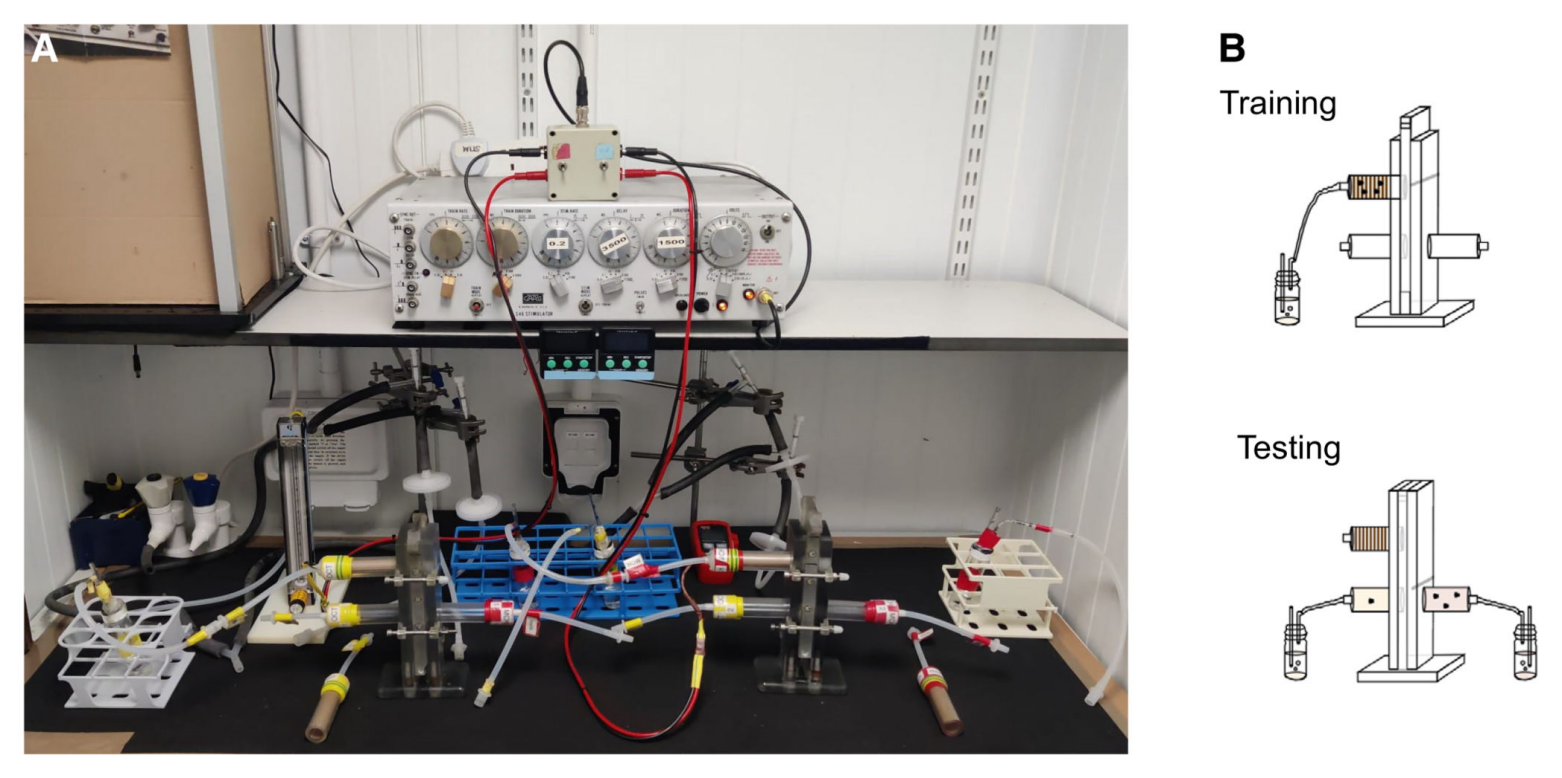
The T-maze and related apparatus for investigating olfactory learning and memory in *Drosophila*. (*A*) The T-maze setup for appetitive and aversive memory used in the Waddell laboratory. (*B*) Flies are trained in the upper portion of the T-maze. Either immediately after training, or at a given time later, the flies can be transferred from the training chamber to the central elevator of the apparatus and transported to the bottom T-maze portion for memory testing, where the flies are given 2 min to choose between the two odors that they experienced during training.

*Although T-mazes are often custom-made and handcrafted from acrylic, plexiglass, and other materials, this protocol provides files and details that allow T-maze manufacture using 3D printing.* Steps 1–40 *detail the assembly of T-mazes and related equipment from 3D-printed parts to generate the basic apparatus* (Fig. 1) *required for aversive olfactory* (Steps 41–74), *appetitive olfactory* (Steps 75–101), *and multisensory aversive and appetitive* (Steps 102–121) *conditioning experiments*.

### Printing Parts for the T-Maze and Fly Collection Device

1. Use the CAD files provided (see Tables 1 and 4) to print the T-maze parts and fly collection device on a 3D printer. Using a different printer model and/or material may affect the precision of the airtight fit around the O-rings; if necessary, adjust design to ensure a good fit of the O-rings.
  *In our model, the parts were printed with the "glossy finish" option (the finish should be as smooth as possible for best results). We found that the VeroUltraClear material was better than VeroClear when performing multisensory experiments*.

### Preparing T-Maze Parts for Assembly

#### Cleaning

**Figure 2:**
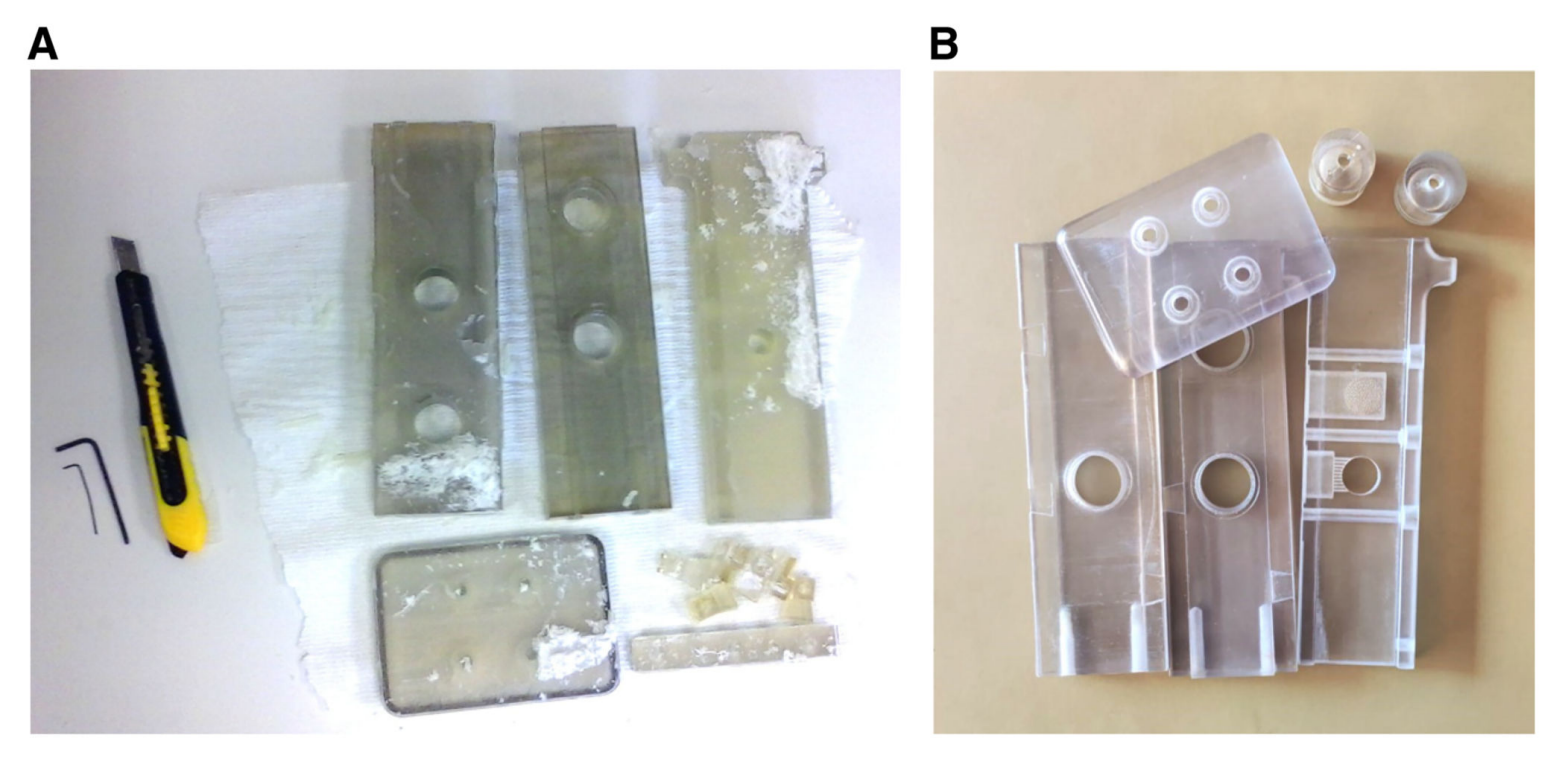
Printed T-maze parts. Parts for the T-maze can be 3D-printed and assembled in-house. (*A*) Example of printed parts and tools used for cleaning. (*B*) Example of printed parts after cleaning.

Figure 2 *depicts parts before and after cleaning*.

2 Using a knife or hex keys, remove the bulk of the support material from the printed parts. You can also use a spare printed part as a plastic scraper.
3 Use the 0.70-mm hex key or a wire to clean out the air holes in the center elevator part.
4 Remove any remaining support material with soap, tap water, and a plastic bristle scrubbing brush. Leave to dry.

#### Tapping

**Figure 3:**
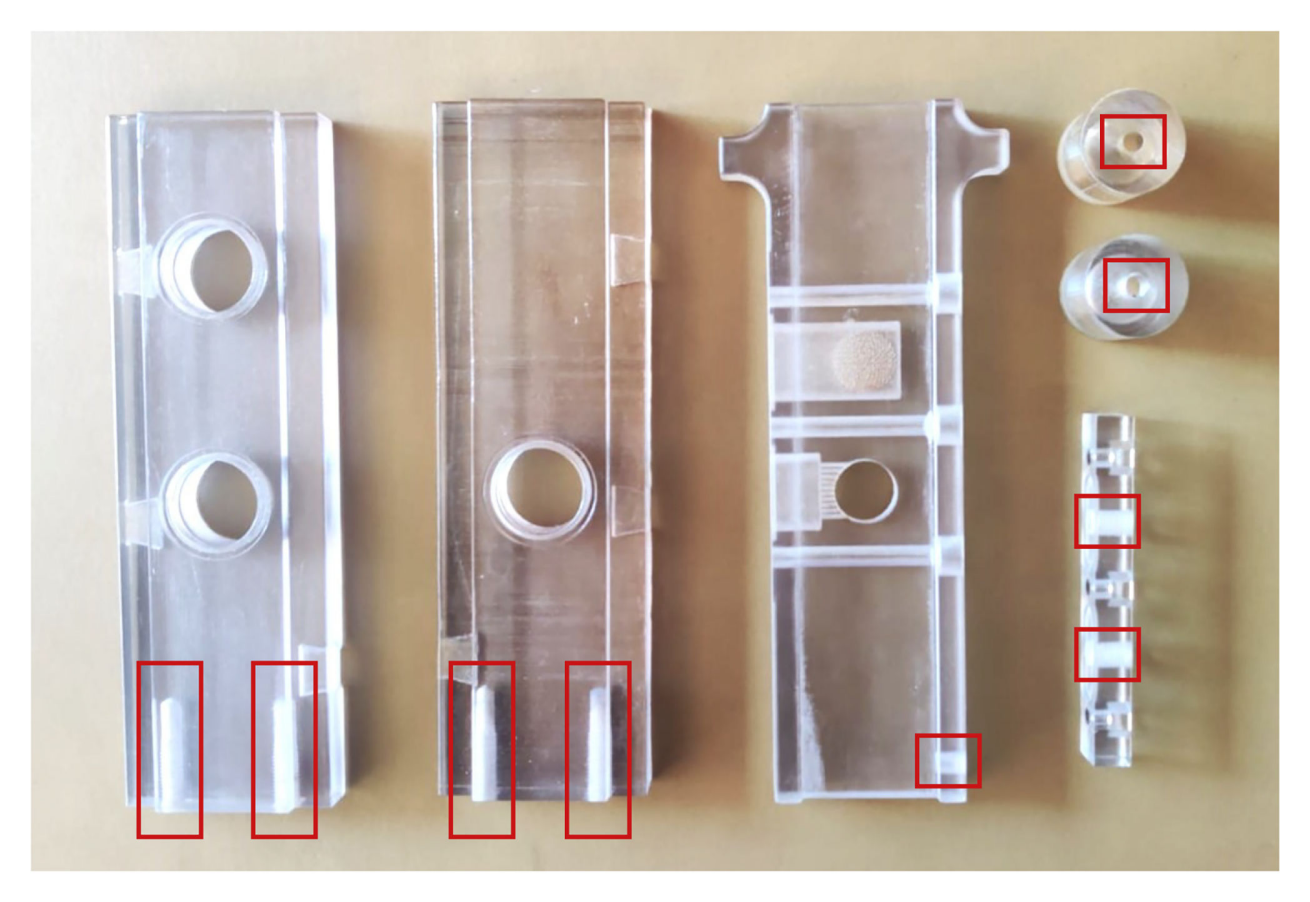
Tapping printed T-maze parts. When assembling the T-maze, screw threads must be created in the predrilled holes of the printed T-maze parts. Tapping locations are depicted (using red rectangles) on the elevator, side walls, and tube caps.

Figure 3 *shows tapping locations*.

5 Tap the two printed pilot holes at the bottom of the left and right walls with a thread tap (e.g., an M6 gauge).
6 Tap the side of the elevator with the M6 tap. Be careful not to strip the threads. Start with a tapered or plug tap, and then switch to a bottoming tap to reach the end of the pilot hole. Stop turning when the tap reaches the end of the pilot hole!
7 Tap the two pilot holes of the tubing adaptor mount with the 1/4-in 28-thread tap. Tap tube caps with the same thread tap.

### Assembling the T-Maze and Tubes

**Figure 4:**
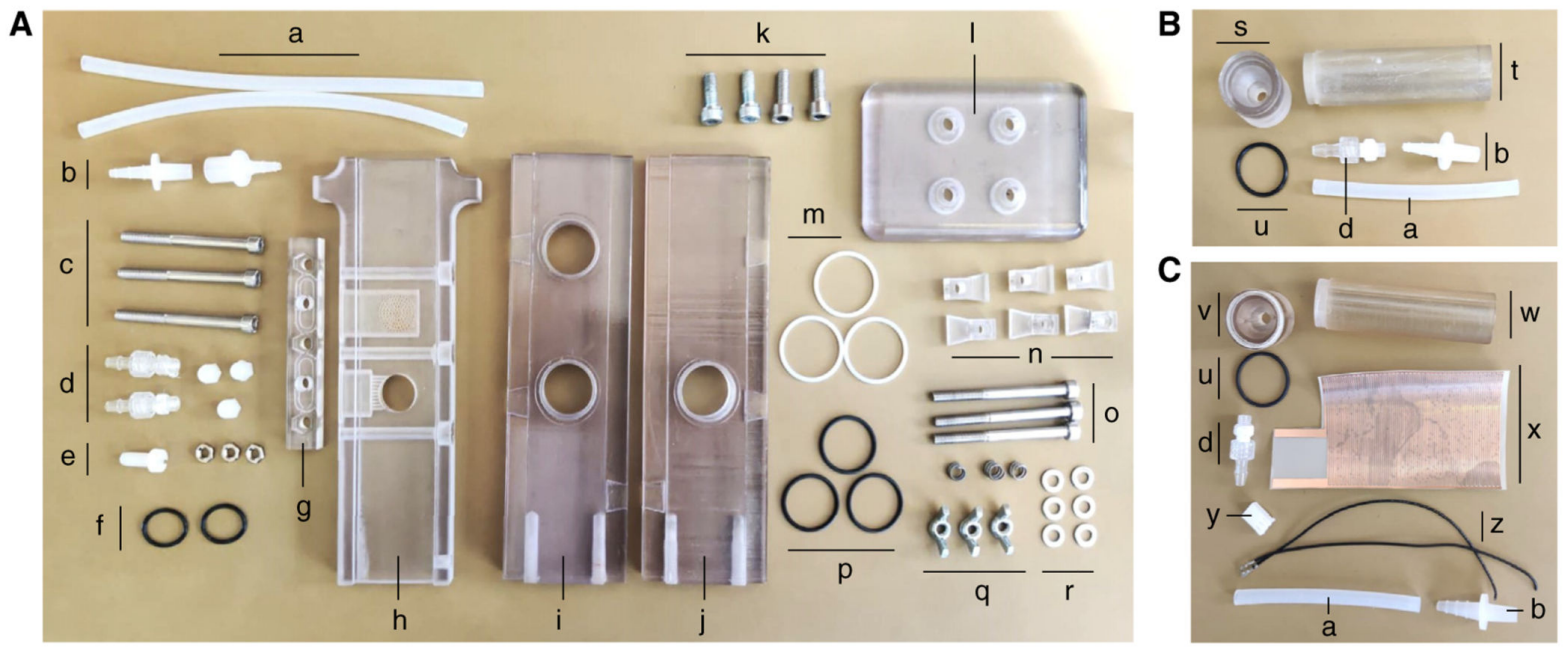
Components of the T-maze and its tubes, ready for assembly. (*A*) T-maze parts include the Silicone tubing (*a*), Kartell quick connectors (*b*), M5 × 60-mm socket screws (*c*), Luer connectors (*d, left*), M5 nylon dome nuts (*d, right*), M6 nylon screw (*e, left*), M5 nuts (*e, right*), BS016 O-rings (*f*), tubing adaptor mount (*g*), elevator (*h*), left wall (*i*), right wall (*j*), M6 × 25 socket screws (*k*), base (*l*), BS022 O-rings (*m*), clamp parts (*n*), M5 × 60-mm socket screws (*o*), BS020 O-rings (*p*), springs (*q, top*), M5 wingnuts (*q, bottom*), and M5 washers (*r*). (*B*) Tube parts include the tube cap (*s*), tube (*t*), and BS019 O-ring (*u*). (*C*) Shock tube parts include the shock tube cap, with holes (*v*); shock tube, with a crescent-shaped gap (*w*); copper grid (*x*); Molex female connector housing (KK) (*y*); and precrimped leads (KK) (*z*).

*T-maze parts are depicted in* Figure 4.

### T*-*Maze Elevator

**Figure 5:**
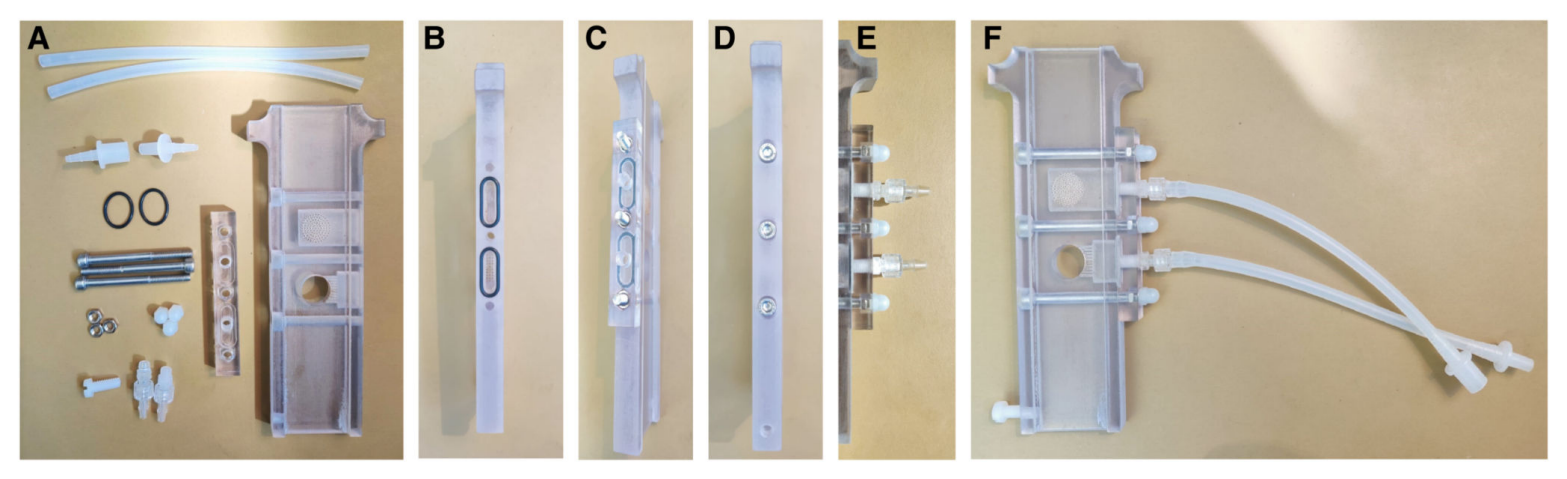
Assembling the T-maze elevator. (*A*) T-maze elevator parts, ready for assembly. (*B*) O-rings are inserted on the side of the elevator. (*C*–*E*) Elevator with tubing mount. (*F*) T-maze elevator, fully assembled.

*The T-maze elevator parts are shown in* Figure 5.

8 Insert the BS016 nitrile O-rings into the grooves on the side of the elevator. Attach the tubing mount using the M5 × 60-mm screws and hex nuts, followed by the nylon dome nuts to prevent scratching.
9 Attach the Luer connectors. Use thread seal tape and tighten connectors with a wrench.
10 Attach the M6 nylon screw into the M6 tapped hole of the elevator. Connect the silicone tubing and Kartell connectors.
  *This tubing connects to the vacuum system in the behavior room and is required to draw air and odorants through the machine*.

### T-Maze Side Walls and Base

**Figure 6:**
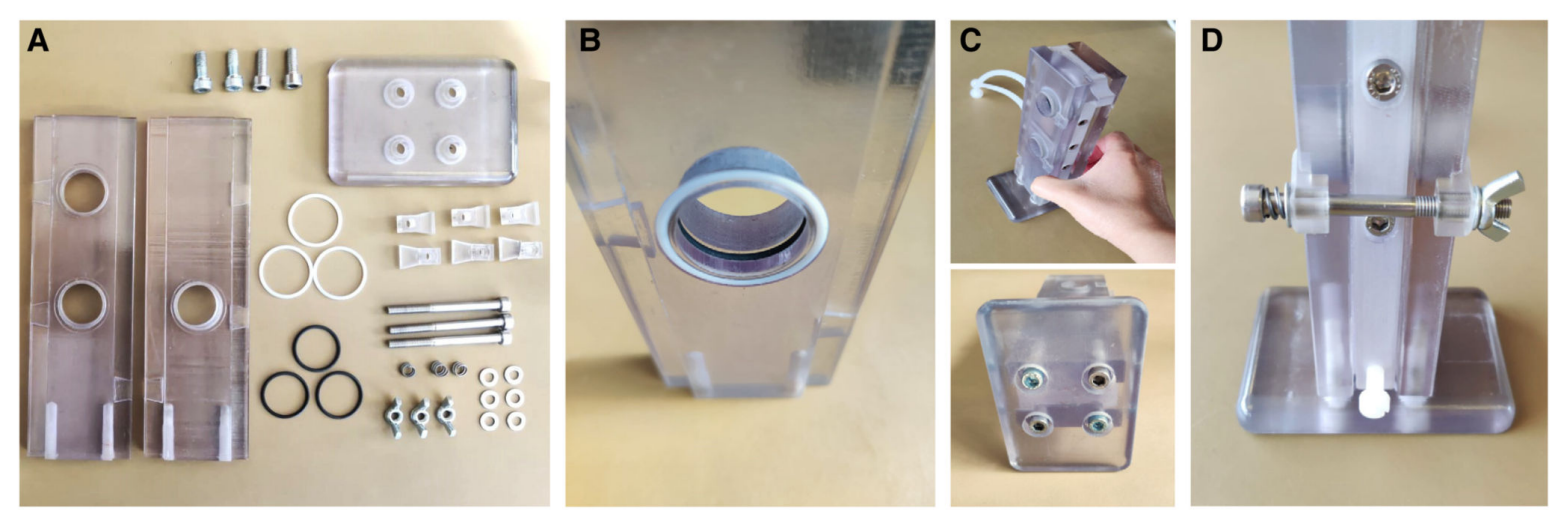
Assembling the T-maze. (*A*) Parts for T-maze side walls, base, and clamps, ready for assembly. (*B*) Example of nitrile and Teflon O-rings inserted in place on one of the side walls. (*C*) Joining T-maze walls, elevator, and base. (*D*) Example of a clamp assembled onto the T-maze. Clamp parts listed from *left* to *right*: M5 × 60-mm screw, short spring, M5 washer, left printed clamp part, right printed clamp part, M5 washer, and M5 wingnut.

*The T-maze side walls, elevator, and base are depicted in* Figure 6.

11 Insert BS020 nitrile O-rings (black) into bore grooves of the left and right walls.
12 Insert BS022 Teflon O-rings (white) into the front grooves of the left and right walls. Press the rings in firmly.
13 Put the center elevator part between the two side walls. Hold together in the middle and attach the base using the four M6 screws.
14 Assemble the three clamps at the sides of the T-maze.

### Assembly of Standard Tubes

**Figure 7:**
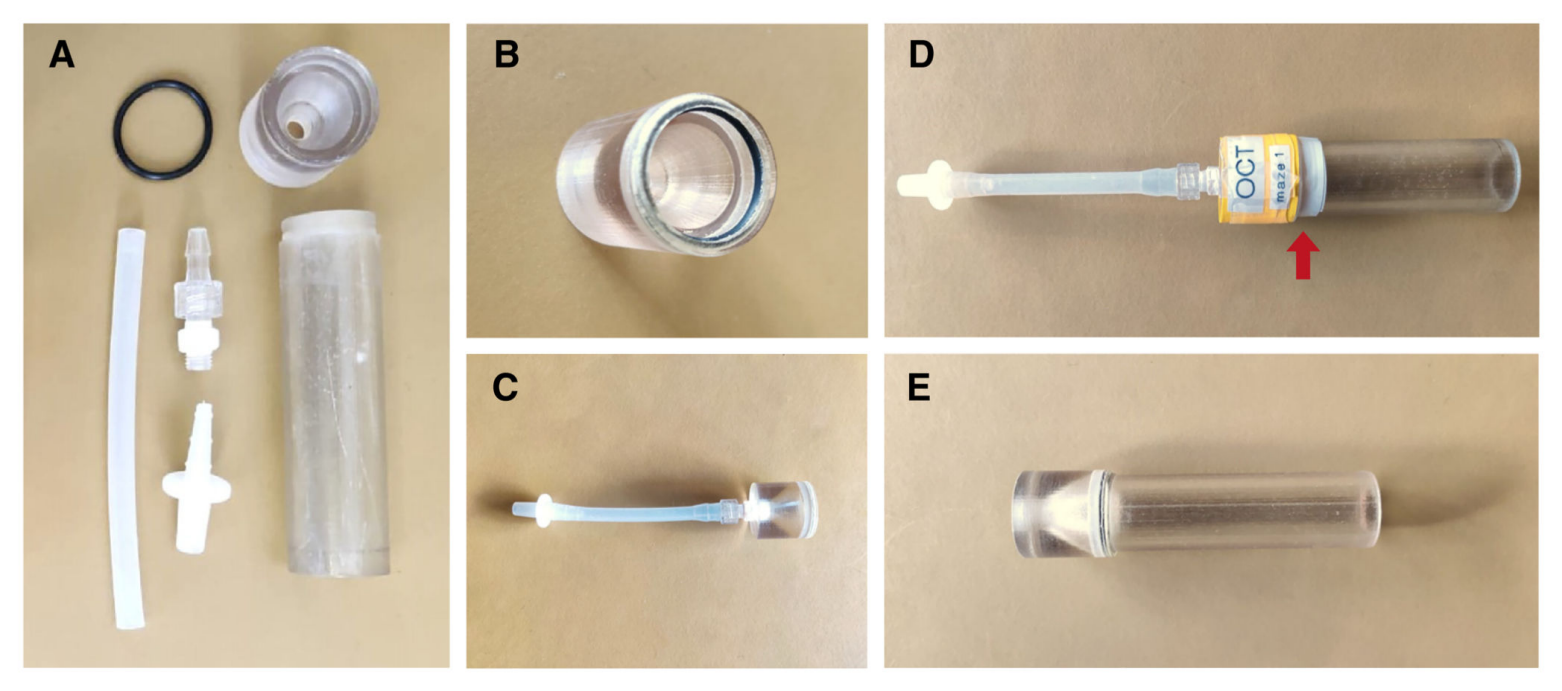
Assembling standard T-maze tubes. These tubes can be used as appetitive training tubes (when lined with sugar paper), loading tubes, and testing tubes. (*A*) Parts for the standard tube. (*B*) Nitrile O-ring fitted inside tube cap. (*C*) Tube cap with Luer connector, silicone tubing, and quick connector attached. (*D*) Example of a testing tube for OCT. The red arrow indicates the position of the electrical tape used for additional sealing. (*E*) Example of a loading tube.

*These can be used as appetitive training tubes, loading tubes, and testing tubes. Parts for these tubes are shown in* Figure 7. *If an appetitive conditioning experiment is carried out using two odors, two appetitive training tubes, one loading tube, and two testing tubes are required for each T-maze. If carrying out an aversive conditioning experiment, omit the two appetitive training tubes*.

15 Assemble the tube cap by inserting a BS019 nitrile O-ring into the groove inside the cap and attaching a Luer connector using thread seal tape.
16 Fit the tube cap tightly on the tube.
17 For training and testing tubes only:
  - Attach a 10-cm piece of Nalgene 50 silicone tubing with a quick connector to the Luer connector on the cap.
  - Label training and testing tubes so that their use is dedicated to a particular odor; for experiments involving two odors, e.g., 3-octanol (OCT) and 4-methylcyclohexanol (MCH), one training and testing tube pair will be labeled OCT, and the other pair will be labeled MCH. Additional labels on training tubes indicating the presence/absence of reinforcer (e.g., sugar) are helpful.
    *Electrical tape can be used to label the tubes in different colors, and we typically cover the joint between the tube and tube cap as an additional measure against air leaks* (Fig. 7D, *red arrow)*.

### Assembly of Aversive Training Tubes

**Figure 8:**
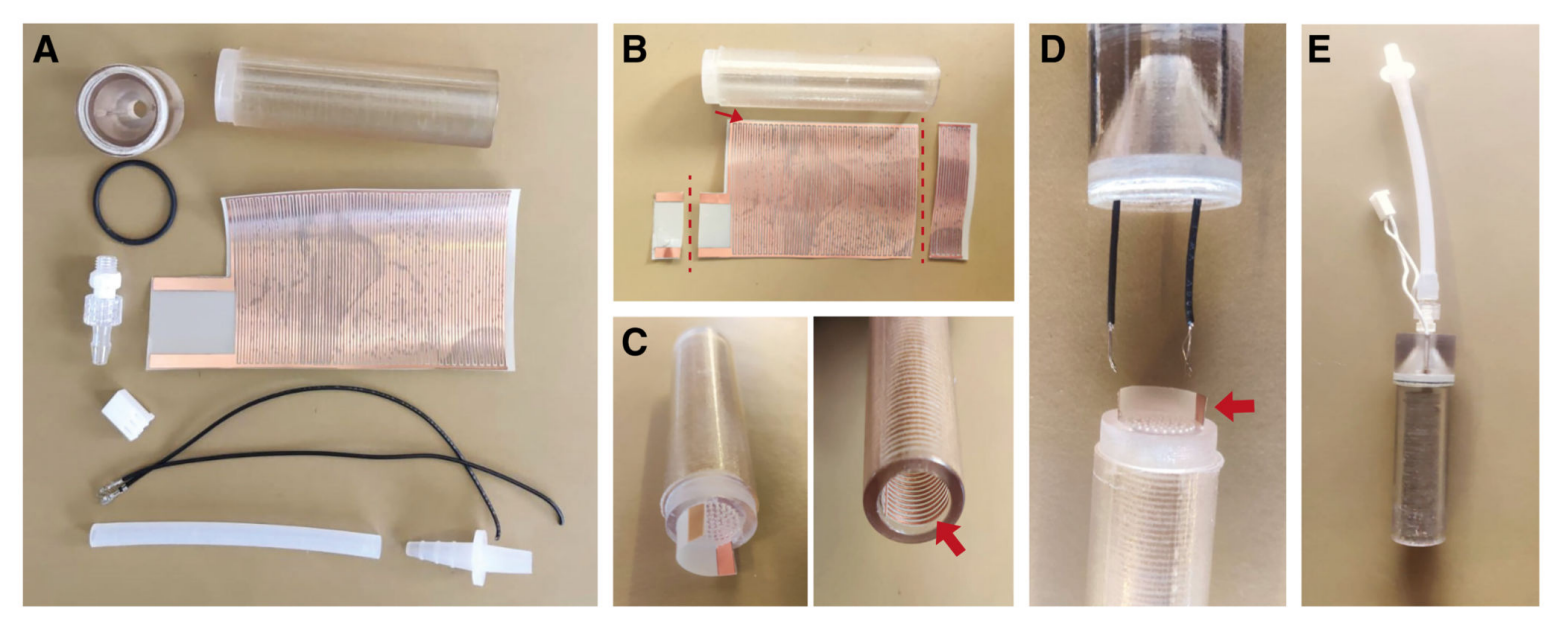
Assembling aversive training T-maze tubes. These grid-lined tubes are used to shock flies during aversive training. (*A*) Parts for aversive training tube. (*B*) Grid cut to size. Dashed lines indicate cut lines. Red arrow points to the edge where a narrow strip of insulating material remains. (*C, left*) Grid rolled and inserted into the tube. (*Right*) Red arrow showing grid fit. (*D*) Leads are inserted through the tube cap to be soldered with the grid contacts. (*E*) Example of assembled aversive training tube with silicone tubing, quick connector attached.

Figure 8 *shows aversive training tube parts. For aversive conditioning experiments, two aversive training tubes are required (in addition to the standard tubes described above)*.

18 To assemble the aversive training tube cap (with two holes for wiring), insert a BS019 nitrile O-ring into the groove inside the cap and attach a Luer connector using thread seal tape.
19 To assemble the aversive training tube, cut a copper grid to size as shown in Figure 8B. Leave a small strip of insulating material on the side edges to avoid short-circuiting. Shorten the grid length to match the tube size, such that the last of the coils remains within the tube when inserted. Roll the grid so that the copper surface is on the inside and insert it into the aversive training tube, allowing the contacts to protrude through the slit. Ensure no space remains between the tube’s inner wall and the grid’s back surface.
20 Insert precrimped leads through the assembled tube cap, strip the ends of the leads, and solder onto the contacts.
  *Before soldering, cut the contacts as short as possible* (Fig. 8D, *red arrow). This allows the tube cap to fit without damaging the connections*.
21 Install the tube cap tightly on the tube, taking care not to twist or damage the contacts. Add the silicone tubing and quick connector. Use hot glue to ensure an airtight seal around the leads and cap. Attach the connector housing onto the leads.
  *For improved durability of the connections, use heat-shrink tubing to protect and cover the leads and connector housing (not shown in figure images)*.
22 Label the tube with the designated odor, as described in Step 17.
  *Electrical tape can be used to label the tubes in different colors designating different odors. When using the tape, cover over the line joining the tube and tube cap as an additional measure against air leaks*.

### Assembling the Switch Box

*A switch box is required for aversive training. This switch box splits a BNC input (coming from the stimulator) to two pairs of outputs, with a gating switch to allow current to pass through. The box connects to shock tubes via banana plug cables listed in* Table 2. *Parts required for assembling the switchbox are listed in* Table 3.

23 To assemble the switch box, connect the signal terminal of a female BNC connector to two on/off toggle switches, splitting the signal.
24 Wire the ground terminal to two black banana plug sockets. Wire each toggle switch output to individual red banana plug sockets.
  *The completed circuit splits the input signal, provides manual on/off control, and acts as a BNC-to-banana plug adaptor. The splitting of the signal can be done after the toggle switches, in which case only one switch would be needed*.

### Assembling Odor Delivery Vials

**Figure 9:**
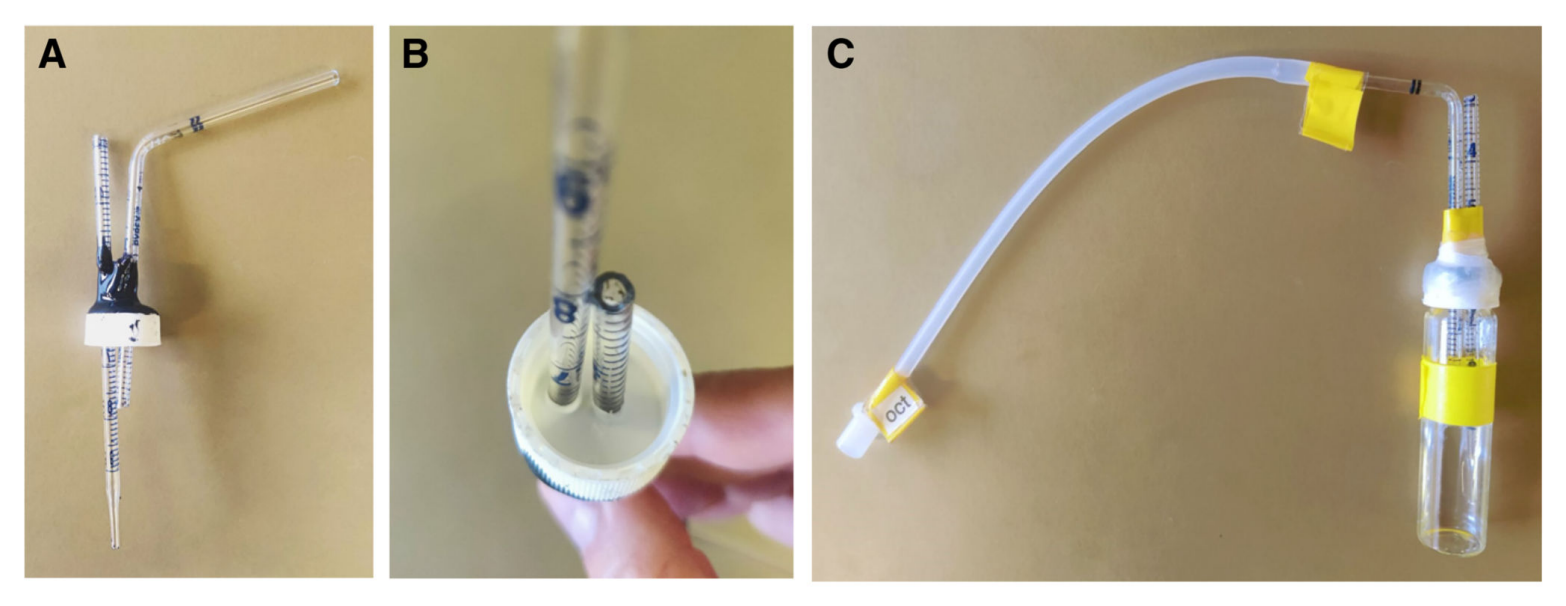
Assembling odor delivery vials. These vials are attached to training and testing T-maze tubes, allowing for odor-infused air to travel through the tubes and T-maze that is connected to a vacuum source. (*A*,*B*) Odor delivery vial cap. (*C*) Assembled odor delivery vial for OCT.

*An odor delivery vial cap and assembled odor delivery vial are shown in* Figure 9.

*If the conditioning experiment involves using two odors, two odor delivery vials will be required for each T-maze. Odor delivery vials and caps can be reused across experiments if the designated odor use is not changed*.

25 Drill two adjacent holes (each hole 4 mm wide) in the screw cap with the electric drill.
26 Using the glass cutter, break a 1-mL glass pipette into two equal pieces and heat-polish the broken ends.
27 Heat one half-pipette at its midpoint over a Bunsen burner and, after heating, bend it into an “L.” Cool to room temperature.
28 Using ethanol for lubrication, slide the end of the straight (half) pipette through one hole in the cap until midlength. Push the end of the L-shaped pipette through the other hole so that the bottom protrudes out slightly.
29 After inserting the pipette, seal both entries in the cap with V9500 epoxy. Leave to dry (~1 d). Cover cap with Parafilm for additional sealing and airtightness.
30 Attach a 30-cm piece of Nalgene 50 silicone tubing with quick connectors to the L-shaped pipette.
  *This tubing will connect to the training or testing tube*.
31 Label the cap with the designated odor. Attach to a 30-mL glass vial and label with the same odor.

### Setting Up the T-Maze for Experiments

#### Preparing the Bench Setup

**Figure 10:**
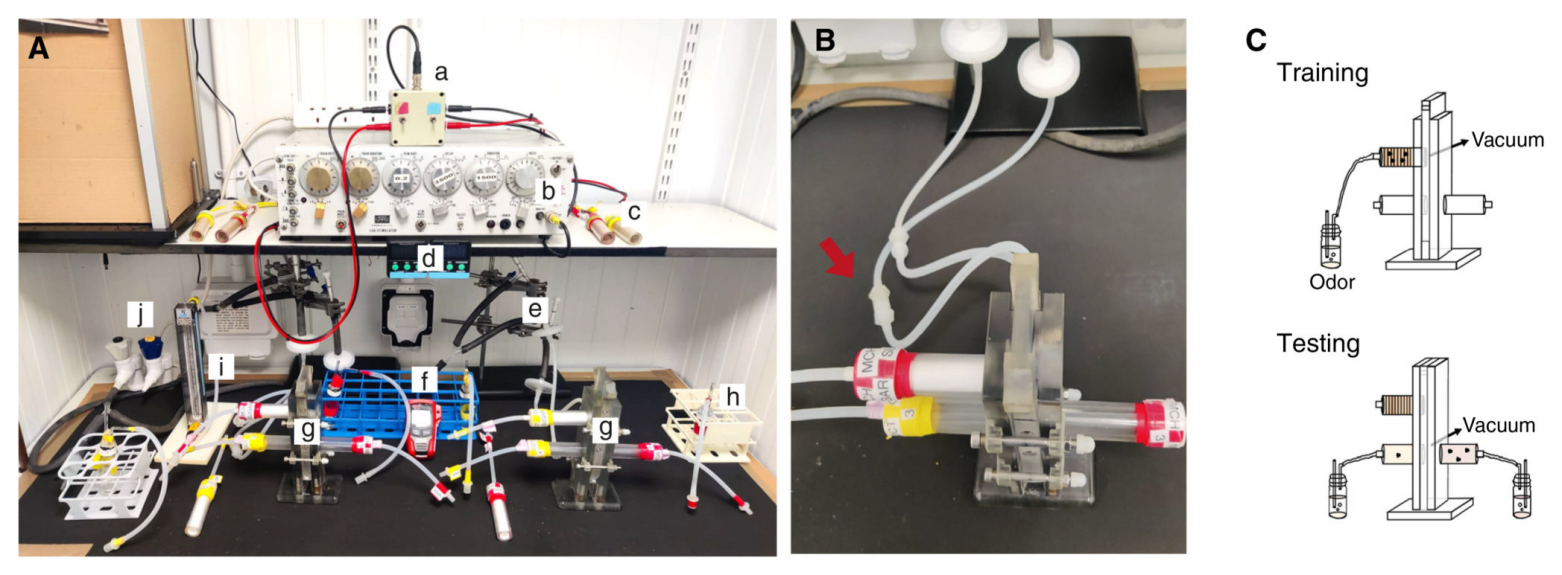
*Drosophila* olfactory conditioning. (*A*) Apparatuses for aversive and appetitive conditioning experiments include switchbox with leads and BNC cable (*a*); S48 stimulator (*b*); aversive training tubes (*c*); timers (*d*); Inline needle valve and Millipore filter, with thick rubber tubing attached to vacuum port (*e*); temperature and humidity data logger (*f*); T-mazes, with appetitive training tubes and testing tubes assembled (*g*); odor delivery vial (*h*); flowmeter (*i*); and vacuum port (*j*). (*B*) Quick connectors (red arrow) are used to hook up the T-maze to the tubing leading to the vacuum port. (*C*) Schematic for training and testing configurations for the T-maze. (*Top*) Training: The designated odor delivery vial is connected to the top training tube, and the elevator is in register to draw odor-suffused air through the training tube. (*Bottom*) Testing: Different odor delivery vials are attached to either test arm. The elevator is now in register with the two testing tubes, so the two odors used in training are being drawn into either arm of the T-maze. Flies can move freely into either T-maze arm.

*Items in the bench setup are depicted in* Figure 10. *Use shelving to store equipment used during the experiments*.

32 Prepare a behavior chamber/room where temperature and humidity are stable and, ideally, can be adjusted. This is required for both appetitive and aversive conditioning experiments. Specific temperature and humidity settings are described in Step 48.
33 Place the T-mazes on a foam mat (we use two layers of neoprene sheeting) covering a hard surface bench.
34 Ensure access to a vacuum port close to the T-mazes.

#### Preparing the Collection Tubes

**Figure 11:**
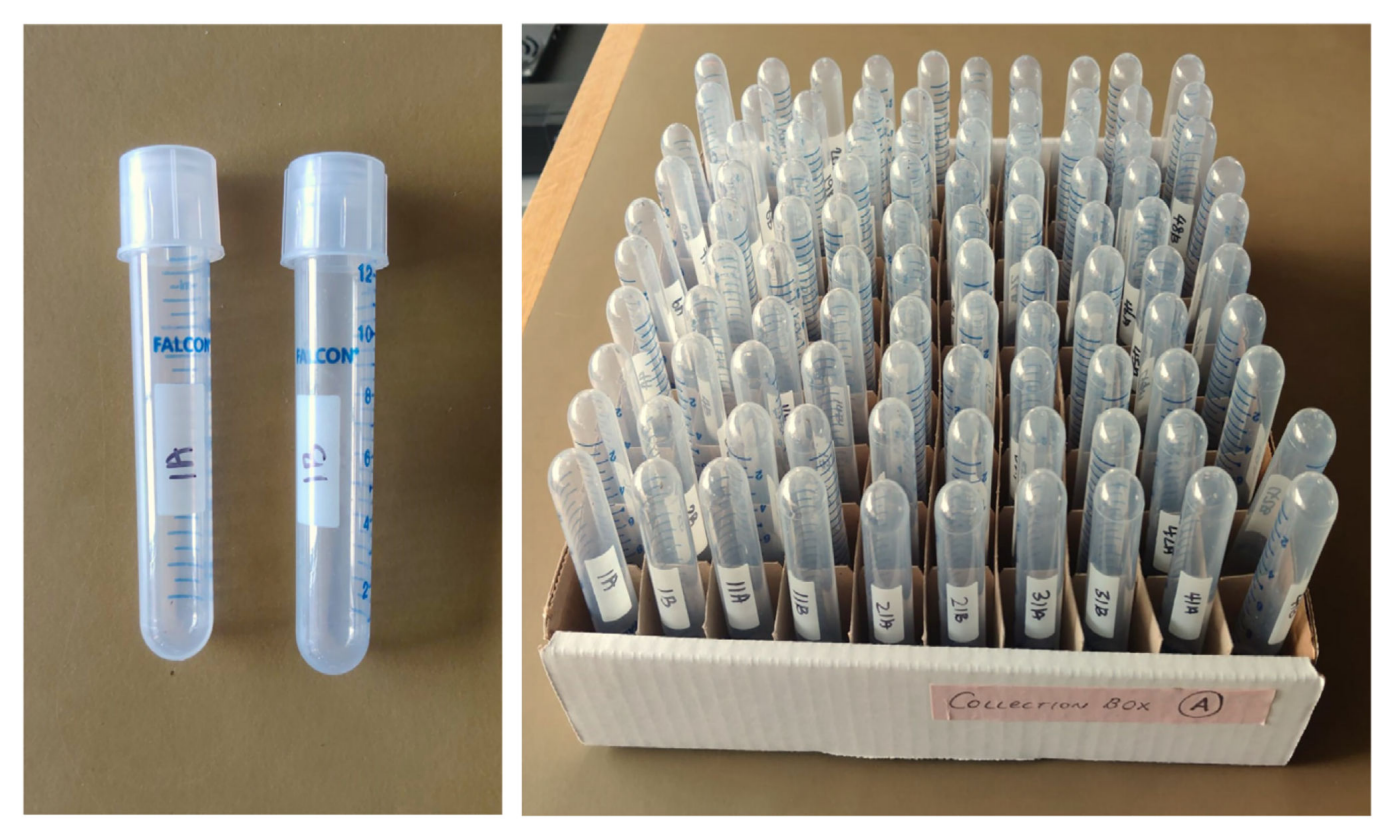
Fly collection tubes. Following aversive or appetitive memory testing, flies are transferred from each testing arm of the T-maze into these collection tubes.

*Collection tubes* (Fig. 11) *are used to collect flies from the T-maze after testing. They are reusable across experiments*.

35 Assemble a box of collection tubes (14-mL polystyrene round-bottom tubes). Label each collection tube pair with a number and letter (i.e., a or b) to represent each arm of the T-maze.

#### Miscellaneous

36 Acquire three timers, a pen, and a sheet to record experimental conditions.
  *To record experimental conditions, we use the template available at* https://github.com/CNCBWaddellTmaze/TMaze/releases/tag/TMaze-v1.0.

### Cleaning and Assembling the Fly Collection Device

**Figure 12:**
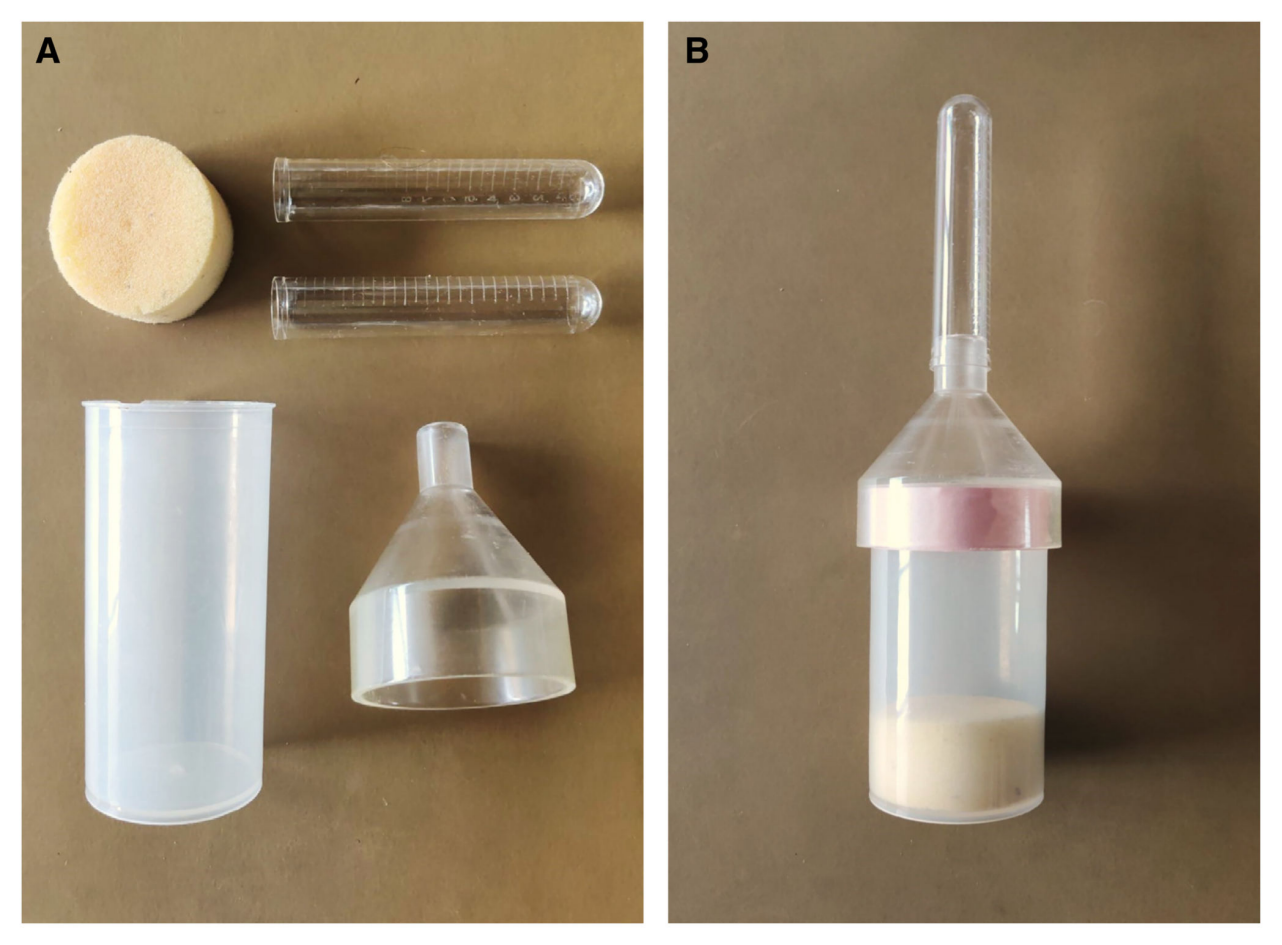
Fly collection device. This collection device is used to separate flies into standard group sizes in preparation for behavior experiments. (*A*) Parts for the fly collection device. (*B*) Assembled fly collection device.

*A deconstructed and an assembled fly collection device are shown in* Figure 12.

*For T-maze behavior experiments, the flies need to be separated into groups of around 100 without using anesthetics. The collection device described below permits flies to be efficiently aliquoted into specific group sizes*.

37 Scrape the support material off the funnel and collection cylinder. Clean the parts using water and soap. Allow to dry.
38 Put a layer or more of electrical tape around the top edge of the cylinder such that the funnel can be securely attached but still be removable.
39 Fit a circular cut sponge in the base of the collection cylinder.
40 Set aside two 14-mL polystyrene round-bottom tubes to be used with the apparatus. Ensure the tubes fit securely on the funnel tip and can be readily attached and removed (use electrical tape to tighten the fit if necessary).

### Aversive Olfactory Conditioning

*This section provides a detailed description of aversive conditioning where, briefly, flies are trained by pairing a 1-min presentation of one odor with 12 electric shocks then with a 1-min presentation of a different odor without punishment. Aversive memory is subsequently tested by giving the flies 2 min to choose between the two odors experienced during training*.

#### Preparing the Flies

**Figure 13:**
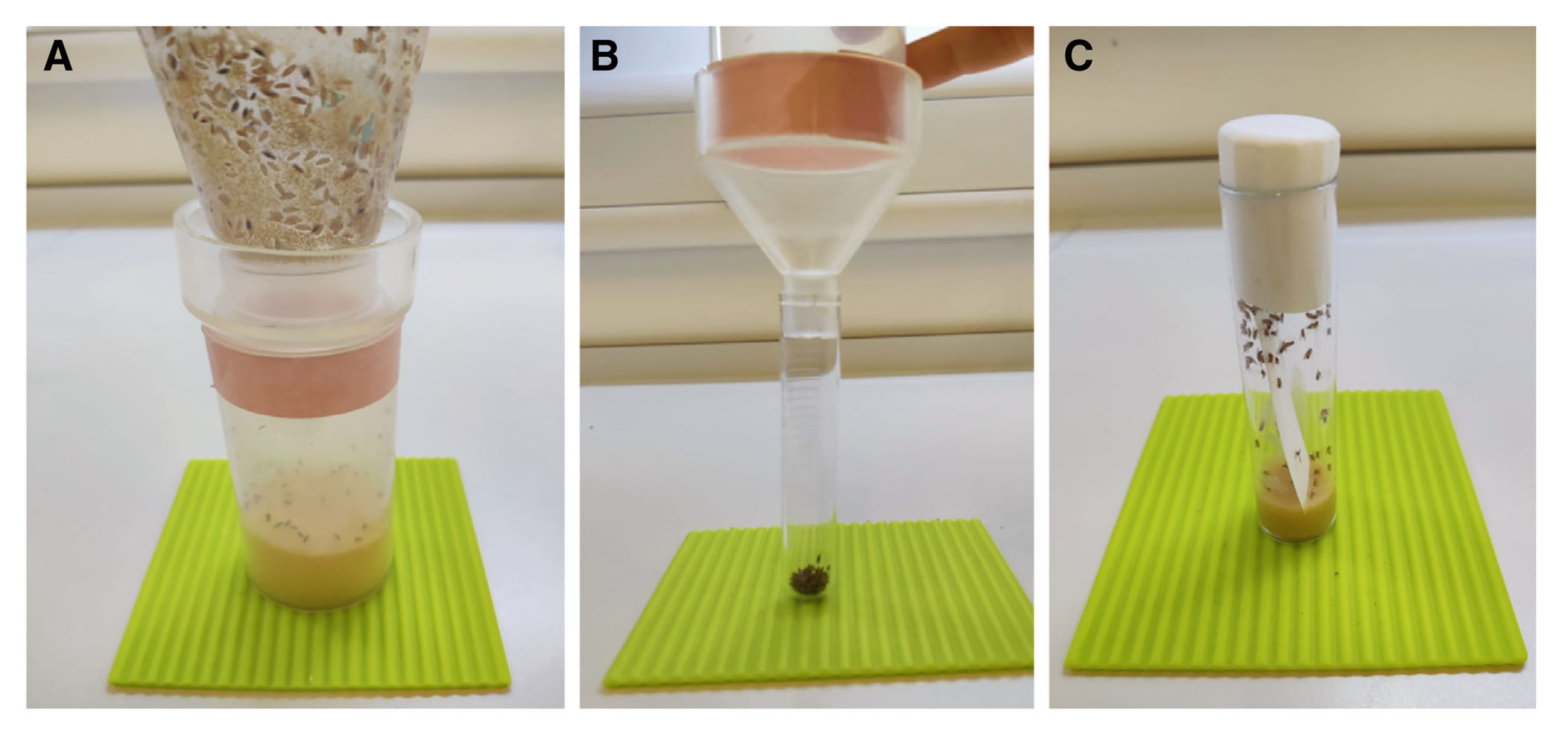
Collecting and aliquoting flies for use in behavior experiments. One day prior to behavioral experiments, flies are divided into standard-sized groups and placed in individual vials. (*A*) Flies are collected in the cylinder using the inverted funnel. (*B*) The funnel is placed back upright on the cylinder and approximately 100 flies are separated into the collection tube attached to the funnel (0.75-to 1-mL mark on the tube). (*C*) The flies in the collection tube are transferred into a fly food vial with a small piece of filter paper.

41 Twelve hours to 24 h before the experiment, place a 5-cm × 2-cm (L × W) piece of Whatman 3MM filter paper into each fly food vial to be used in the experiment (this is to control humidity and provide more surface area for the flies to use). Transfer flies, separated by genotype, to these food vials using the collection device (Fig. 13), following the steps below:
  - Invert the funnel part of the collection device and place it onto the collection cylinder.
  - Dump the flies into the collection cylinder.
  - Turn the funnel upright and place it back onto the cylinder. Attach the collection tube to the end of the funnel.
  - Invert the device to collect approximately 100 flies at the bottom of the tube, aiming for just below the 1-mL mark.
  - Remove the collection tube from the funnel and transfer the flies into a food vial. Plug the vial immediately to prevent flies from escaping.
    *Following these steps, you should end up with approximately 100 flies per vial without the need for anesthetizing them, which can influence their subsequent behavior*.
42 Keep the flies overnight at 23°C, at a relative humidity of 60%, on a 12-h light/dark cycle.
  *Flies used should ideally be 2*–*7 d after eclosion. The commonly used wild-type strain for T-maze olfactory memory experiments is* Canton-S. *Use appropriate genetic controls when testing transgenic flies from different sources, because learning and memory*–*directed behaviors are sensitive to genetic background. For all experiments, we aliquot a few additional pairs of wild-type flies to use in case troubleshooting (e*.*g*., *adjusting odor bias, testing the efficacy of the sugar paper) is necessary*.

#### Diluting the Odors

43 To prepare the odor mixtures, pipette 8 mL of mineral oil into each of the two odor delivery vials (assembly of these vials is described in Steps 25–31). Add 8 µL of OCT to the vial labeled OCT. Add 8 µL of MCH to the vial labeled MCH. Prepare the odor mixtures as close to the start of the experiment as possible.
  *When presented in our T-mazes with our odor delivery setup, naive flies distribute approximately 50:50 between the odors described in Step 43. Experimenters should empirically determine relative odor concentrations producing an equal distribution of naive flies using their own apparatus and chosen odor pairs. Before starting each day's experiment, ensure that naive flies do not show a significant bias toward one of the odors, as this will weaken the performance index after training. See Troubleshooting*.
44 Place the designated odor delivery caps on each vial.
45 Vortex vials for 20 s to mix the odor solutions.

#### Setting up the T-Maze for Aversive Conditioning Experiments

**Figure 14:**
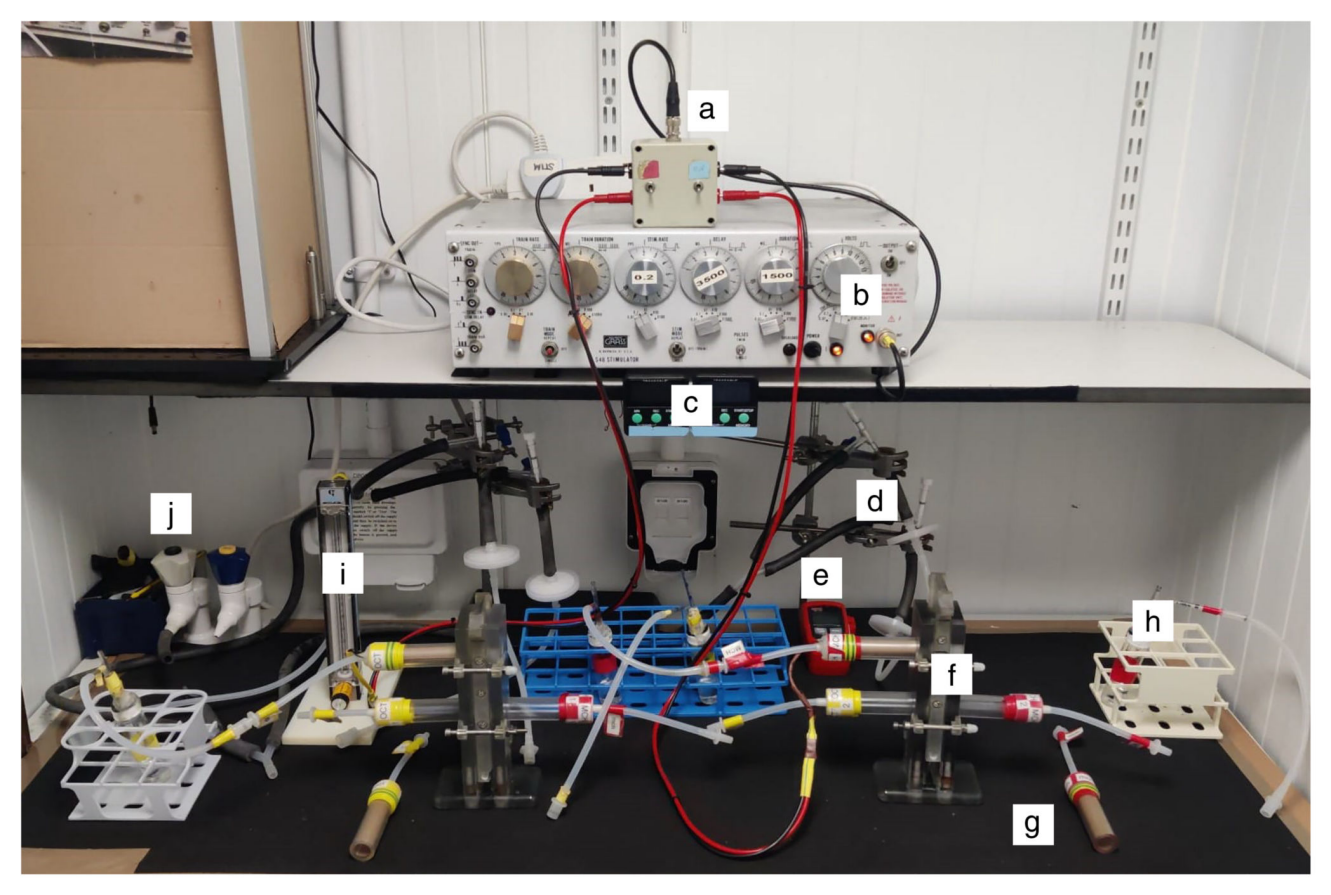
Aversive conditioning setup. Apparatus for aversive conditioning experiments consists of switch box with leads and BNC cable (*a*); S48 stimulator (*b*); timers (*c*); inline needle valve (to regulate airflow) and Millipore filter (to block debris), with rubber tubing attached to vacuum port (*d*); temperature and humidity data logger (*e*); T-maze, with shock training tube and testing tubes assembled (*f*); mock training tube (*g*); odor delivery vial (*h*); flowmeter (*i*); and vacuum port (*j*).

46 Prepare the bench setup and the T-maze for experiments, as described in Steps 32–36 (Fig. 14).
47 Gather tubes required for aversive conditioning:
  - two “shock” aversive training tubes (copper grid connected to leads), one for each odor
  - two “mock” aversive training tubes (copper grid not connected), one for each odor
  - two testing tubes, one for each odor
  - one loading tube (empty tube for transporting flies)
    *Quantities listed are per one T-maze, for the conditioning experiments using two odors. Tubes should be labeled clearly and not be mixed between odors. The tubes are assembled as described in* Steps 15–22.
48 Set the behavior room temperature to 23°C unless temperature-sensitive alleles/transgenes are used. For aversive training, the behavior room humidity is crucial (it should not be <55%;~70% is optimal).
49 Ensure the T-maze is clean and free of dead flies (wipe with a damp tissue).
  *If necessary, T-mazes can be washed with detergent, water, and ethanol, but it is critical to rinse them well with water to eliminate any lingering odor. Washed mazes should be left to dry and air for several days*.
50 Connect the tubing on the back of the T-maze to the vacuum system in the behavior room.
51 Position the T-maze elevator in the middle of the machine so that the vacuum pulls air through the training tube. Attach the flowmeter to the tubing on the training tube and adjust the airflow with the inline needle valve so that the vacuum pulls air through the training tube at a rate of 750 mL/min.
  *See Troubleshooting*.
52 Reposition the T-maze elevator so that the vacuum pulls air through the testing port. Attach the flowmeter to one of the testing tubes and connect the other testing tube to its respective odor delivery vial. Adjust the needle valve so the airflow through each testing tube is at 750 mL/min.
  *See Troubleshooting*.

#### Electric Shock Delivery Stimulator Settings (for the Grass S48 Stimulator)

53 Set the stimulator to the following settings: Stim. Rate: 0.2 PPS, Delay: 3.5 sec, Duration: 1.5 sec, Volts: 90 V, Stimulus Mode: “Repeat.”
  *This ensures that each 1-min odor training session is paired with 12 electric shocks*.
54 Connect the stimulator to the switch box and turn on the stimulator (keep the switch box off).

#### Aversive Training

55 Transfer the flies from a food vial into the shock training tube (for odor A, OCT) and attach it to the upper port of the T-maze, with the elevator positioned for training. Keep thumb over the tube opening to prevent flies from escaping during the transfer.
56 Make sure electric leads from the switch box are connected to the shock training tube, but do not switch on the box.
57 Connect odor A (OCT) to the shock training tube and immediately turn on the switch box. Start the timer and wait for 1 min. The flies in the shock tube will receive 12 electric shocks during the 1-min presentation of odor A (OCT).
58 At the end of 1 min, turn off the switch box and disconnect odor A (OCT). Leave the flies in the tube for 45 sec.
59 Gently knock the T-maze on its side to move the flies to the end of the shock training tube. Remove the tube with flies and transfer them to the mock training tube (for odor B, MCH). Attach the tube to the upper port of the T-maze.
60 Next, connect odor B (MCH) to the mock training tube for 1 min, but do not turn the shock switch on.
61 After 1 min of odor B (MCH), disconnect the odor and leave the flies in the tube for another 30 sec before moving them.
  *If testing immediate memory or "learning," omit* Steps 62 and 63 *and go directly to* Step 64.
62 If testing later memory, transfer the flies from the training tube back into their original food vial, where they will remain until testing.
63 For testing, transfer the flies back into the loading tube and attach the tube to the upper port of the T-maze.

#### Testing

64 Slide the elevator of the T-maze so that the elevator hole is in register with the training (or loading) tube.
  *See Troubleshooting*.
65 Knock the flies from the training (or loading) tube into the hole in the elevator by sharply tapping the T-maze on its side. Quickly push the elevator halfway down so that the flies are now confined within the elevator.
  *See Troubleshooting*.
66 Set the timer for 2 min. Connect odor A (OCT) to the left testing tube and odor B (MCH) to the right testing tube.
67 Switch off the overhead lights, push the elevator all the way down so the hole is even with the two testing tubes, and start the timer.
  *See Troubleshooting*.
  *During testing, flies will distribute between the two testing tubes, each tube suffused with either of the previously trained odors. This step should be performed in the dark to eliminate potential phototactic effects from uneven lighting*.
68 After 2 min, raise the elevator to trap the flies in the testing tubes and switch the overhead lights back on.
  *See Troubleshooting*.
69 Disconnect the odors.
70 Carefully tap the machine on its side to knock the flies to the bottom of one testing tube, detach the testing tube, and transfer the flies to a collection tube (e.g., labeled 1a).
  *See Troubleshooting*.
71 Repeat Step 70 with flies in the opposite testing tube, transferring them to a separate collection tube (e.g., labeled 1b). Discard the few flies that remain in the elevator. Visually inspect the distribution of flies in the two collection tubes to spot any obvious odor bias.
  *See Troubleshooting*.
  *In a behavior sheet, note the CS*^*+*^ *odor (for the previously shock-paired odor; in this case, OCT) or CS*^−^ *odor (for the previously non-shock-paired odor; in this case, MCH); note also the genotype tested and any other relevant information (temperature, humidity, odor dilution, age of the flies, etc*.*). The template we use is available at* https://github.com/CNCBWaddellTmaze/TMaze/releases/tag/TMaze-v1.0.

#### Reciprocal Aversive Training

72 Repeat the training and testing (Steps 55–71) with a different population of flies of the same genotype. However, with this group, pair the other odor with the electric shock stimulus (in this example, MCH); pair odor B (MCH) with shock for 1 min, give 45 sec of fresh air, and then odor A (OCT) without shock for 1 min. Use the next pair of collection tubes (e.g., 2a/2b) to collect flies.
  *Reciprocal training is an important feature of the experimental design. It averages and therefore generalizes odor-specific differences and, more importantly, reciprocal training accounts for subtle skew in the distribution of flies that results from an imbalance in the relative concentrations of the odors. For example, a skew away from odor A toward odor B would be amplified if odor A was paired with shock but neutralized if odor B is paired with shock. By averaging the two scores, the naive skew is accounted for. Reciprocal training can be done in parallel with the initial training by using two mazes side by side*. DasGupta and Waddell (2008) *provide an example of experiments that investigate odor-specific differences in learning*.

#### Calculating Memory Performance

73 Count the flies in the collection tubes. Flies can be easily counted when dead! We routinely place them overnight in a −20°C freezer (alternatively, for 15 min in a −80°C freezer).
  *The flies in the CS*^*+*^ *collection tube are those that ran toward the odor previously paired with the electric shock ("wrong choice"). The flies in the CS*^−^ *collection tube are those that ran toward the odor that was not paired with the electric shock ("correct choice"*).
74 Determine the performance index (PI) as follows:
  - Calculate a half-score by subtracting the number of flies in the CS^+^ tube from the number of flies in the CS^−^ tube and dividing by the total number of flies: [(#flies CS^+^) − (#flies CS^−^)]/(total #flies).
  - Determine the half-score in the reciprocal experiment in which the other odor was paired with the electric shock.
  - Average the two half-scores, [(half-score in which OCT was the CS^+^) + (half-score in which MCH was the CS^+^)]/2, to generate a single performance index (PI; *n* = 1).
    *A score (PI) of* −*1 would represent perfect performance with all flies making the appropriate choice following aversive training, whereas a score of 0 would represent the flies distributing evenly between the test tubes and indicates that no memory was expressed. Most published olfactory memory data are* n ≥ *8 per group, where each* n *corresponds to the performance of 200 flies (100 + 100)*.
    *See Troubleshooting*.

### Appetitive Olfactory Conditioning

*This section provides a detailed description of appetitive conditioning where, briefly, hungry flies are trained by giving them a 2-min presentation of one odor followed by a 2-min presentation of a different odor with sucrose reward. Appetitive memory is subsequently tested by giving the flies 2 min to choose between the two odors experienced during training*.

#### Preparing Agar Vials

75 Prepare fly vials with agar base (pour 2–2.5 mL of molten 1% agar solution per vial). The agar will provide a source of hydration for the flies while depriving them of food. When cooled down, wrap the vials (or the box containing the vials) in cling film to prevent the agar from drying and store for up to 1 mo at 4°C. When preparing for experiments, avoid using vials with visible mold or if the agar base has dried up.

#### Preparing “Sucrose” and “Blank” Papers for Appetitive Training Tubes

**Figure 15:**
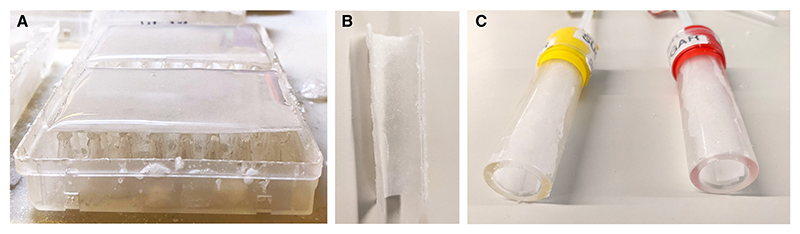
Sucrose paper for appetitive conditioning. Pieces of filter paper coated with sugar need to be prepared for appetitive conditioning experiments. (*A*) A saturated sucrose solution poured on top of filter paper cut to size (for best results, a film of sucrose should be visible). (*B*) Once dried, the paper can be rolled (*C*). The rolled paper is inserted tightly into the training tube. (Avoid rips in the paper when rolling. Excess sucrose will detach from the paper; this is normal. Shake the excess out of the tube.)

76 Cut four 7.5-cm × 5-cm (L × W) Whatman 3MM filter paper rectangles. These papers will provide the lining for the training tubes, and need to ideally be prepared new for each experiment (but can be reused over two to three experiments if necessary).
77 Process the filter papers as follows:
  - Soak two of the rectangle sheets in tap water (distilled water does not work as well). Let them dry completely (few hours to a day). Once dried, roll each paper lengthwise and slide it into an empty training tube. Label the tubes “blank” (or “H_2_O”) and indicate a designated odor for each.
    *These training tubes will be used when flies are exposed to a specific odor without sucrose. Each pair of odors should have two "blank" papers, one for each odor*.
  - Pour the saturated sucrose solution (5.8 M) over two rectangles (a small film of sucrose should be visible on top of the filter paper) and let it dry completely (2–3 d, depending on humidity and temperature). If need be, dry the papers in an incubator at 31°C. Once dried, roll each paper lengthwise and slide it into an empty training tube; label it “sucrose” (or “sugar”), and indicate the designated odor.
    *These training tubes will be used to expose flies to a specific odor in the presence of sucrose. Each experiment should have two "sucrose" papers, one for each odor. See* Figure 15.

#### Preparing the Flies: Food Deprivation

**Figure 16:**
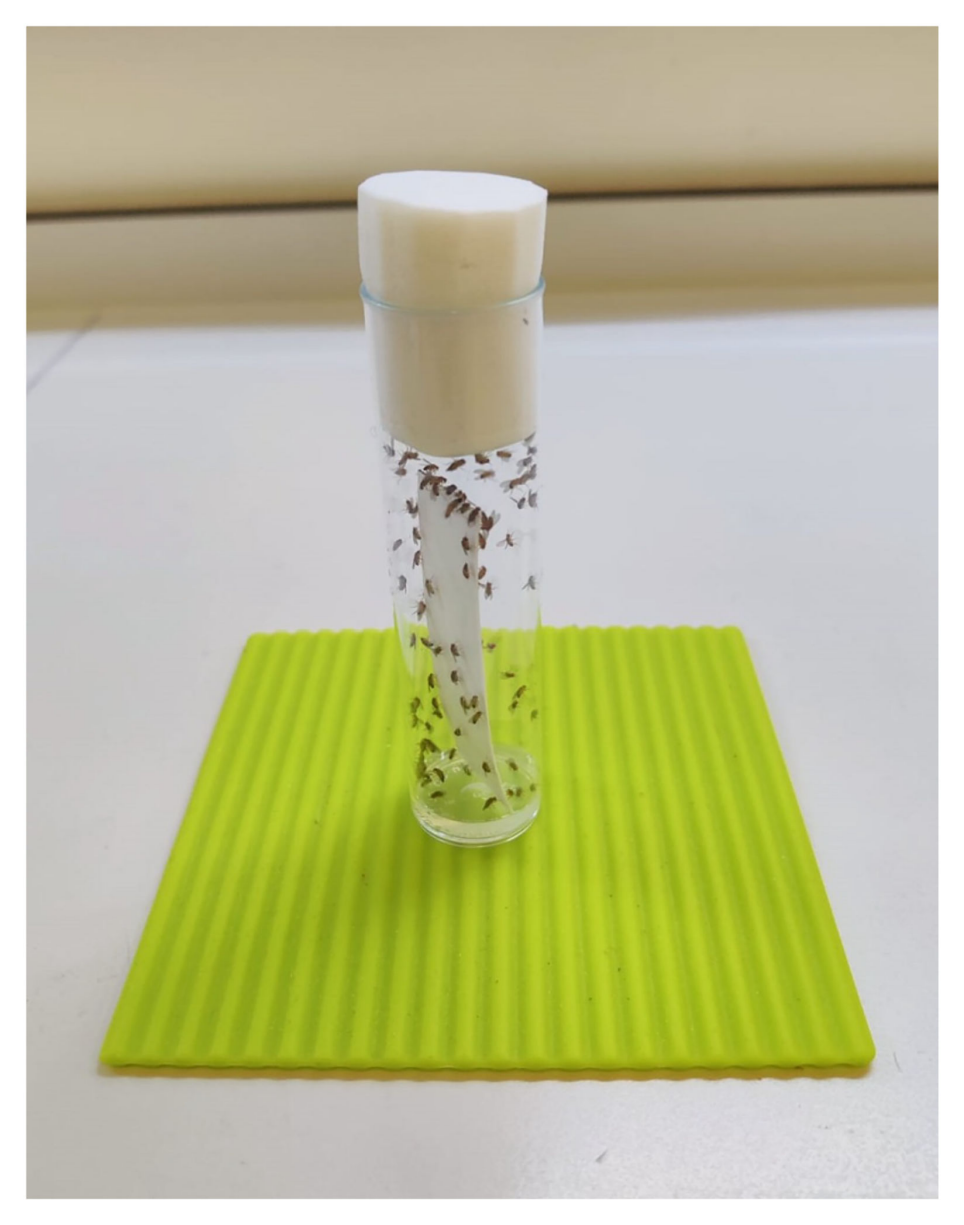
Starving flies prior to appetitive conditioning. Flies are housed in agar-filled vials to food-deprive them before appetitive conditioning experiments.

78 Sixteen hours to 24 h before the experiment, place a 5-cm × 2-cm (L × W) piece of Whatman 3MM filter paper into each agar vial to be used in the experiment (this is to control humidity and provide more surface area for the flies to use). Transfer flies (separated by genotype) to these agar vials (Fig. 16) using the fly collection device, as described in Step 41 (use agar vials instead of fly food vials).
79 Starve flies for 16–24 h (ideally 20–22 h) before training the following day. Keep flies overnight at 23°C, relative humidity 60%, on a 12-h light/dark cycle.
  *Flies used should ideally be 2*–*7 d old (after eclosion). We find that within this age bracket, younger flies are more starvation-resistant; opt for younger flies if you find that many are dead following the starvation period. For all experiments, we aliquot a few additional pairs of wild-type flies to use in case troubleshooting (e*.*g*., *adjusting odor bias, testing the efficacy of the sugar paper) is necessary. A commonly used wild-type strain for T-maze experiments is* Canton-S. *Always use appropriate genetic controls for transgenic flies tested, because learning and memory*–*directed behaviors are sensitive to changes in genetic background*.

#### Diluting the Odors and Setting Up the T-Maze for Appetitive Conditioning Experiments

**Figure 17:**
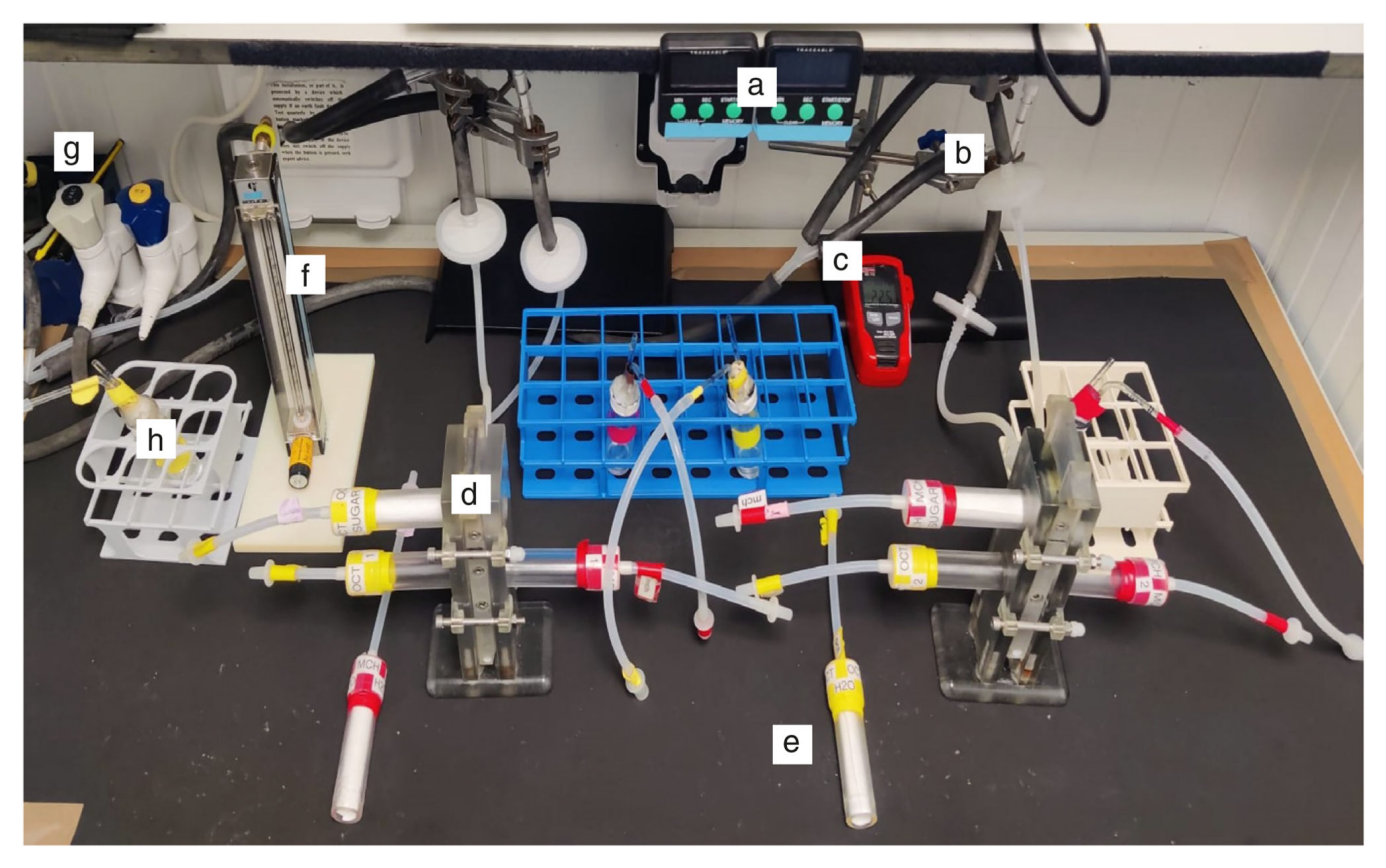
Appetitive conditioning setup. Apparatus for appetitive conditioning experiment consists of timers (*a*); inline needle valve (to regulate airflow) and Millipore filter (to block debris), with rubber tubing attached to vacuum port (*b*); temperature and humidity data logger (*c*); T-maze, with sucrose training tube and testing tubes assembled (*d*); blank training tube (*e*); flowmeter (*f*); vacuum port (*g*); and odor delivery vial port (*h*).

80 Prepare the odors as described in Steps 43–45. Prepare the bench setup, the T-maze, and related equipment as described in Steps 32–36. See Figure 17 regarding equipment setup.
  *The Grass S48 stimulator is not needed for appetitive conditioning*.
81 Gather tubes required for appetitive conditioning:
  - two “sucrose” training tubes, one for each odor (preparation described below)
  - two “blank” training tubes, one for each odor (preparation described below)
  - two testing tubes, one for each odor
  - one loading tube (empty tube for transporting flies)
    *Quantities listed are per one T-maze, for the conditioning experiments using two odors. Tubes should be labeled clearly and not be mixed between odors. The tubes are assembled as described in* Steps 15–17.

#### Appetitive Training

82 Transfer the flies from a food deprivation vial into the blank training tube (for odor A, OCT) and attach it to the upper port of the T-maze, with the elevator positioned for training. Keep thumb over the tube opening to prevent flies from escaping during the transfer.
83 As quickly as possible, connect odor A (OCT) to the blank training tube and start the timer. The flies in the blank training tube will be exposed to odor A (OCT) for 2 min without sucrose.
84 After 2 min, disconnect odor A (OCT) to leave the flies inside the tube receiving room air drawn into the tube by the vacuum pull.
85 Leave the flies in the tube for 30 sec.
86 Gently knock the T-maze on its side to move the flies to the bottom end of the blank tube. Remove the tube with flies and transfer them quickly to the sucrose training tube (for odor B, MCH).
87 Immediately attach the sucrose tube with flies inside to the top port of the T-maze. Immediately connect odor B (MCH) to the sucrose tube and start the timer. Expose the flies in the sucrose training tube to odor B (MCH) for 2 min in the presence of sucrose.
88 After 2 min, disconnect the odor and leave the flies receiving room air for 30 sec.
  *If testing learning or short-term memory, omit Steps 89 and 90 and go directly to Step 91 for testing*.
89 If testing later memory, promptly transfer the flies from the sucrose training tube to the original agar vial, where they will be kept until the time of testing. If testing memory beyond 24 h, transfer the flies to a vial containing food, but subsequently starve them again (16–24 h) before testing.
  *To increase survivability when testing 24-h memory, flies can also be fed briefly (for 30 min) and then transferred to agar deprivation vials until testing. However, note that this may alter memory processing* (Musso et al. 2021).
90 At the time of testing, transfer the flies back into the loading tube and place the tube in the upper port of the T-maze.

#### Testing

91 Slide the elevator of the T-maze so that the elevator hole is in register with the training (or loading) tube.
  *See Troubleshooting*.
92 Knock the flies from the training (or loading) tube into the hole in the elevator by sharply tapping the T-maze on its side and push the elevator halfway down to trap the flies in the elevator.
  *See Troubleshooting*.
93 Set the timer for 2 min. Connect odor A (OCT) to the left testing tube and odor B (MCH) to the right testing tube.
94 Switch off the overhead lights, push the elevator all the way down so the hole is even with the two testing tubes, and start the timer.
  *See Troubleshooting*.
    *During testing, flies will distribute between the two testing tubes, each tube suffused with either of the previously trained odors. This step should be performed in the dark to eliminate potential phototactic effects from uneven lighting*.
95 After a 2-min testing period, raise the elevator to trap the flies in either testing tube. Switch the overhead lights back on.
96 Disconnect the odors.
97 Carefully tap the machine on its side to knock the flies to the bottom of one testing tube, detach the testing tube (e.g., odor A), and transfer the flies to a collection tube (e.g., labeled 1a).
  *See Troubleshooting*.
98 Repeat Step 97 with flies in the opposite testing tube (e.g., odor B), transferring them to a separate collection tube (e.g., labeled 1b). Discard the few flies that remain in the elevator. Visually inspect the distribution of flies in the two collection tubes to spot any obvious odor bias.
  *See Troubleshooting*.
    *In a behavior sheet, note the CS*^*+*^ *odor (for the previously sucrose-paired odor; in this case, MCH) or CS*^−^ *odor (for the previously non-sucrose-paired odor; in this case, OCT); note also the genotype tested and any other relevant information (temperature, humidity, odor dilution, age of the flies, etc*.*)*.
    *The template we use is available at* https://github.com/CNCBWaddellTmaze/TMaze/releases/tag/TMaze-v1.0.

#### Reciprocal Appetitive Training

99 Repeat the training and testing steps (Steps 82–98) with a different population of flies of the same genotype. However, with this group, pair the other odor with sucrose (in this example, OCT): Pair odor B (MCH) without sucrose for 2 min, give 30 sec of fresh air, and then expose to odor A (OCT) with sucrose for 2 min. Use the next pair of collection tubes (e.g., 2a/2b) to collect flies.
  *See note about reciprocal training in* Step 72.

#### Calculating a Performance Index

100 Count the flies in the collection tubes. Flies can be counted when dead after placing them overnight in a −20°C freezer (alternatively, for 15 min in a −80°C freezer).
  *The flies in the CS*^*+*^ *collection tube are those that ran toward the odor previously paired with sucrose (*“*correct choice*”*). The flies in the CS*^−^ *collection tube are those that ran toward the odor that was not paired with sucrose (*“*wrong choice*”*)*.
101 Determine the performance index (PI) as follows:
  - Calculate a half-score by subtracting the number of flies in the CS^+^ tube from the number of flies in the CS^−^ tube and dividing by the total number of flies: [(#flies CS^+^) − (#flies CS^−^)]/(total #flies).
  - Determine the half-score in the reciprocal experiment in which the other odor was paired with the sucrose.
  - Average the two half-scores, [(half-score in which OCT was the CS^+^) + (half-score in which MCH was the CS^+^)]/2, to generate a single performance index or PI (*n* = 1).
    *A score of 1 would represent perfect performance with all flies making the appropriate choice following appetitive training, whereas a score of 0 would represent the flies distributing evenly between the test tubes. Most published olfactory memory data have* n≥*8 per group, where each* n *corresponds to the average performance of around 200 flies (100 from initial training, 100 from reciprocal training)*.
      *See Troubleshooting*.

### Aversive and Appetitive Multisensory Conditioning

*This next section describes the necessary adjustments of the olfactory conditioning paradigms so that specific odors and colors (visual stimuli) can be associated together with sucrose reward or with electric shock punishment* (Okray et al. 2023; Thiagarajan et al. 2022). *Because flies are innately phototactic, the experiments require additional attention to deal with two sources of innate bias: odor and color preference. Relevant troubleshooting notes are included throughout*.

#### LED Assembly

**Figure 18:**
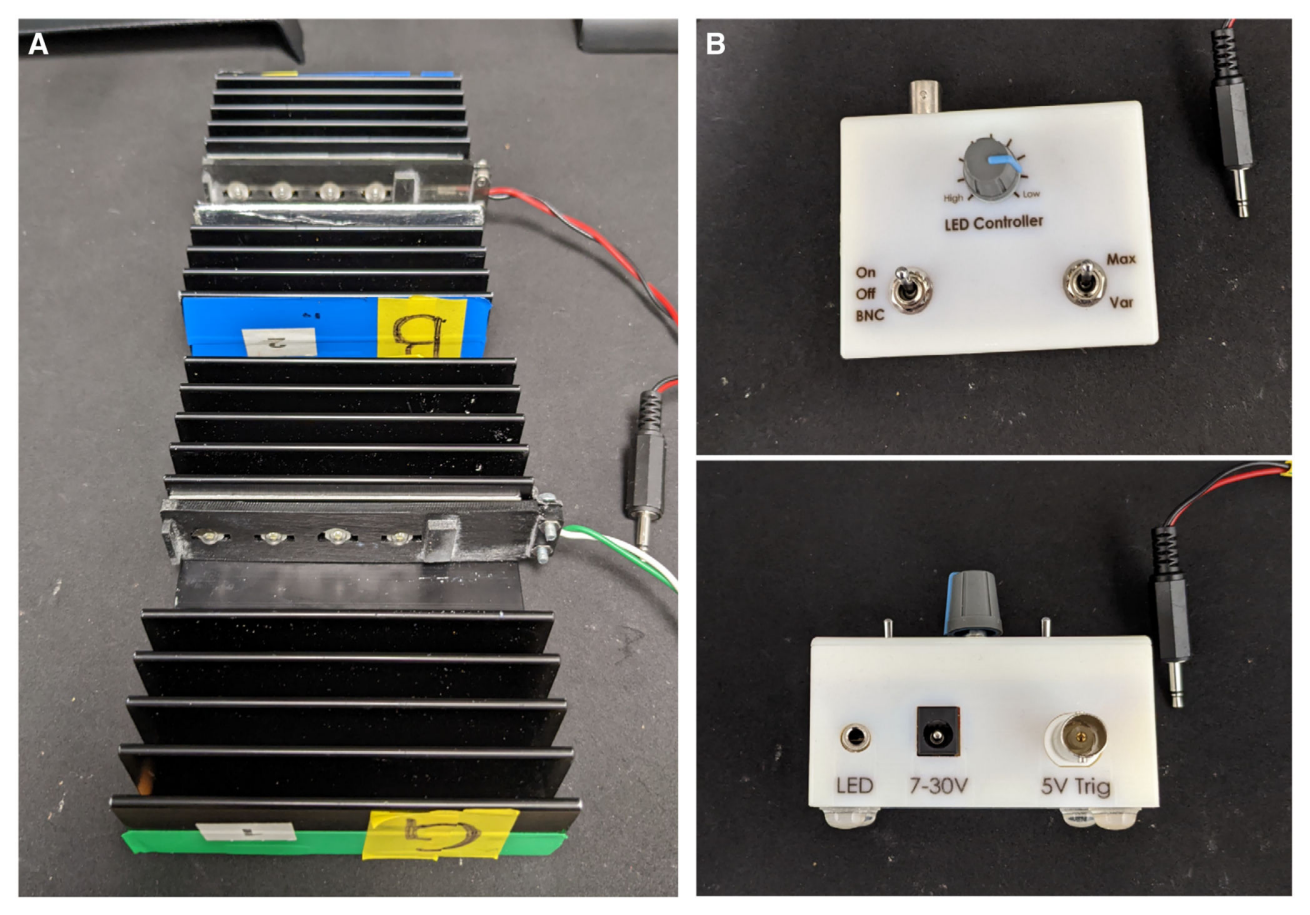
Assembling the LED units. These LED units, composed of heatsinks housing green or blue LEDs, allow for the presentation of color stimuli to flies during multisensory conditioning. (*A*) Blue (*top*) and green (*bottom*) LEDs assembled in the heatsinks. (*B*) Front (*top*) and side (*bottom*) views for the LED controller and its connection from heatsinks.

102 Solder four green LEDs in series on an aluminum PCB. Use a small amount of thermal paste between the LED body and the PCB to help with heat transfer.
103 Solder the brown wire of the LED connection cable to the “+” solder pad on the PCB. Solder the white wire to the adjacent unmarked solder pad. This cable will be used to power the LEDs via the LED controller and its power supply.
104 Attach the PCB (with LEDs) to the heatsink via double-sided heatsink tape. The heatsink is important for the dispersion of heat generated by the LEDs; make sure the PCB is well attached to the heatsink to avoid LED power degradation and failure.
105 Using the M3 screws and nuts, further secure the PCB to the heatsink using a 3D-printed cover to prevent the PCB from dislodging and to keep the cables in place. See Figure 18.
  *The LED controller provides the LEDs with a constant current (up to 700 mA). The LEDs can be turned on/off manually or triggered with a 5-V signal via a BNC cable. The current can be manually varied to adjust LED brightness*.

#### Preparing the Flies

106 Prepare the flies according to Steps 41 and 42, or Steps 75, 78, and 79, depending on whether aversive or appetitive conditioning experiments will be conducted.
  *Add four to six extra pairs of vials containing wild-type flies (around 100 per vial). These flies will be needed when adjusting for color/odor bias at the start of the experiment*.

#### Setting Up the T-Maze for Multisensory Learning Experiments

**Figure 19:**
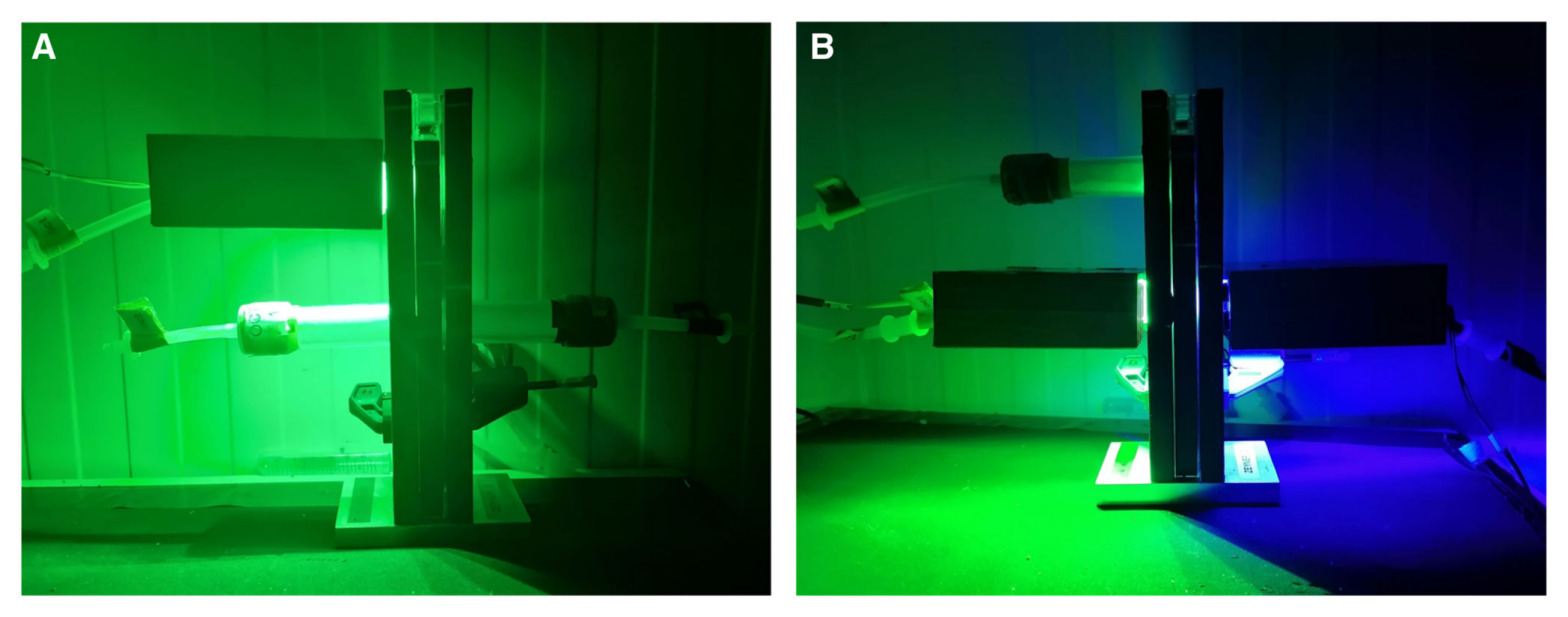
Multisensory conditioning T-maze setup. This setup enables the simultaneous presentation of odor and color stimuli during aversive and appetitive training and testing protocols. (*A*) Training configuration with a green LED unit. (*B*) Testing configuration with green LED (*left*) and blue LED (*right*) units.

*The multisensory learning T-maze setup is shown in* Figure 19.

107 Prepare the odors, the bench setup, and the T-maze for experiments, as described in Steps 43–54 or Steps 80 and 81, depending on whether aversive or appetitive conditioning experiments will be carried out.
108 Ensure that for each T-maze in the setup, a pair of heatsinks (one green, one blue) is available.
109 To keep the quality of the transmitted light/color consistent between training and testing, line the test tubes with the same material as the training tubes (i.e., “mock” electric grid for aversive conditioning and “blank” filter paper for appetitive conditioning).

#### Adjusting for Color Bias

*In multisensory learning experiments, flies are always handled under overhead red lights in the behavior room. This is to reduce the exposure of flies to unspecific color stimuli. The T-maze is covered in matte black, self-adhesive (removable) sheeting to minimize color interference*.

110 Transfer the flies to a dark environment (e.g., inside an opaque black bag) 30 min before experiments.
  *Flies should be removed from darkness only when training or testing, during which they must be handled under an overhead red light*.
111 Load untrained wild-type/control flies from one vial onto the T-maze, with the elevator hole in register with the loading tube.
112 Place the heatsink with green LEDs on one testing arm of the T-maze, and the heatsink with blue LEDs on the other arm. Set one odor to be associated with green (e.g., odor A [OCT]) and one to be associated with blue (e.g., odor B [MCH]). Turn on the LEDs. Colored light should be delivered continuously. Ensure that the heatsinks sit evenly on the training tubes and are positioned as closely as possible to the T-maze side walls (Fig. 19). Consistent placement of the heatsinks/LEDs is important for delivering the color stimuli in a reproducible way across trials. To avoid wobbling of heatsinks, you can choose to place a thin layer of silicone tape on the training and testing tube caps.
  *See Troubleshooting*.
    *Learning and memory performance may vary depending on the specific pairing of colors and odors; we find that pairing OCT with green and MCH with blue works well*.
113 Turn off the overhead red light. Push down the elevator until flies are released into the T-maze arms.
114 After 2 min, lift the elevator, turn off the LEDs, remove the flies, and place them in the collection tubes. Check for reasonably even distribution (by eye or by counting).
115 If necessary, repeat with another set of flies, adjusting the intensity of the green or blue LEDs until flies distribute equally between the two colors.
  *See Troubleshooting*.
    *Ensure that odor bias is resolved before adjusting for color bias*.

#### Multisensory Training

116 Transfer the flies to a dark environment (e.g., inside an opaque black bag) for 30 min before training starts.
117 Follow the training steps for aversive conditioning (Steps 55–63 and 72) or appetitive conditioning (Steps 82–90 and 99). Implement the following changes:
  - Pair the CS^+^ (and CS^−^) odor presentation with a color by placing the relevant LED heatsink on top of the CS^+^ (and CS^−^) odor tubes, and turning on the LEDs simultaneously with odor delivery.
    *The weight of the heatsink on the tube can affect the tube seals and airflow, so measure the airflow in the training tubes when the heatsinks are attached*.
      *See Troubleshooting*.
      *If testing learning or short-term memory, omit Steps 118 and 119 and go directly to Step 120 for testing*.
118 If testing later memory, promptly transfer the flies from the training tube to the original vial and keep the vials in the dark until testing.
119 At the time of testing, transfer the flies back into the loading tube and assemble onto the T-maze.

#### Testing

120 Follow the testing steps for aversive conditioning (Steps 64–71) or appetitive conditioning (Steps 91–98). Implement the following changes:
  - When the flies are in the elevator, place the heatsinks on top of the respective testing tubes. Connect the odors to the testing tubes and turn on the LEDs before pushing the elevator into testing position.
    *As noted previously, verify the airflow in the testing tubes when the heatsinks are attached. See Troubleshooting*.

#### Calculating Memory Performance

121 Count and calculate memory performance as described for aversive conditioning (Steps 73 and 74) or appetitive conditioning (Steps 100 and 101).

## Related Information

### Control Assays for Sensory Acuity and Locomotion

Olfactory conditioning involves flies associating odors with either electric shock punishment or sugar reward (see **Introduction: Twists to Classical Conditioning of Adult *Drosophila*** (Okray and Waddell 2024). In both cases, memory is measured as behavioral performance—whether the flies avoid or approach the arm of the T-maze infused with the appropriate odor. It is therefore critical to determine that the memory defect does not result from an inability to sense odors, sugar, or shock or to move well enough to perform the memory task (defective locomotion might reduce performance in all acuity tests). The acuity assays are straightforward and quick to perform. Olfactory and shock acuity are measured in the T-maze in the same way as memory and are therefore also good “task-relevant” controls in addition to reporting the appropriate locomotor capability.

#### Olfactory Acuity

To test olfactory acuity, untrained flies are given the choice between a diluted odor (as used in conditioning) and air bubbled through mineral oil in the T-maze. Flies are loaded into the machine as if testing memory and transported in the elevator to the T-maze choice point. After a 2-min choice period, they are trapped in either arm, removed, and counted. A performance index is calculated as the number of flies in the air arm minus those in the odor arm divided by the total number of flies. Most odors used in conditioning are naturally aversive at the concentrations used. One can also vary odor concentration to test relative acuity more rigorously.

#### Shock Reactivity

The shock acuity assay is performed and quantified similarly. Untrained flies chose between a tube containing an electrified grid and a tube containing a nonelectrified grid. After a 2-min choice period, they are trapped in either arm, removed, and counted. A performance index is calculated as the number of flies in the nonelectrified arm minus those in the electrified arm divided by the total number of flies. One can also vary the intensity of the electric shock to test relative acuity more rigorously.

#### Sugar Acuity

Sucrose acuity has been measured in different ways over the years (Tempel et al. 1983; Schwaerzel et al. 2003; Keene et al. 2006; Wu et al. 2020). One method closely resembles the appetitive conditioning paradigm. Here, flies starved for 16–24 h are given 2 min to choose between an arm of the T-maze containing a “sucrose” tube and the other containing “blank.” Both papers are prepared as described in Steps 76 and 77 of the protocol. A preference index (PI) is calculated as the number of flies in the “sucrose” minus that in the other arm, divided by the total number of flies. Keeping the overhead light on in the behavioral room and having airflow running through the tubes greatly enhances the preference index in wild-type flies (Wu et al. 2020).

Alternatively, one can use a variant of the taste preference test, frequently used in gustation studies (Marella et al. 2006). Flies are starved for 16–24 h, and taste preference is assayed on quadrant plates, in which two opposing quadrants contain 1% agarose ± 100 mM sucrose. Approximately 60 flies are placed on the plate and allowed to explore the agarose quadrants for 5 min, at which time they are recorded using a digital camera and video software. The number of flies on each quadrant is manually counted at the 5-min time point. A sucrose preference index is calculated as PI = [(#flies on sucrose quadrants) − (#flies on agarose)]/(total #flies).

#### Data Analysis and Statistics

It is critical that the behavioral data are subjected to the appropriate statistical analysis. If performed incorrectly, irrelevant differences might be considered important, and important differences might be missed. Because of the inherent variability of behavior, small differences are unlikely to be judged as significant if the number of samples is too low. Likewise, low sample numbers can sometimes yield statistical differences that disappear as the number of samples increases. Most published olfactory memory data are at least *n*≥8 for each genotype (across three or more different days of experiments), and the scores are displayed as the mean PI ± standard error of the mean (SEM). We routinely perform statistical analyses using GraphPad Prism (GraphPad Software). Unpaired *t*-tests (or their nonparametric version) are used to compare two relevant groups. One-way ANOVA (or Kruskal–Wallis, for nonparametric analysis) followed by Dunnett’s multiple comparisons test (for planned comparisons to a specific group) or Tukey’s multiple comparisons test (for comparison between different genotypes) are used as post-hoc tests to compare data between groups. The one-sample *t*-test (or its nonparametric version) can be used to test for a difference between a theoretical mean of 0 (i.e., a significant difference from 0 means that flies either avoid or approach the odor). For further discussion of statistics in biology, see Zar (2010).

## Troubleshooting

***Problem (Steps 43, 71, and 98):*** Fly distribution is abnormally skewed during testing due to an underlying, naive odor bias, independent of learned preference.

***Solution:*** Odor concentration may not be ideal. Change the odor concentration (±1–4 µL in 8 mL of mineral oil) until 50/50 distribution between odors is achieved. Increasing the concentration of an odor (within this ±1- to 4-µL range) will make the odor more aversive. DO NOT change the concentration of the odors in the middle of an experiment; correct for any odor bias at the beginning of the experiment using spare wild-type flies (Steps 41 and 42, 78, and 79).

***Problem (Steps 43, 71, and 98):*** Fly distribution is abnormally skewed during testing due to an underlying, naive odor bias, independent of learned preference.

***Solution:*** There could be residual odor or debris in the tubes. Clean the tubes with a damp tissue. If the tubes are very dirty, ethanol or odorless soap can be used to thoroughly clean them. Make sure to rinse well with tap water and leave to dry.

***Problem (Steps 51 and 52):*** T-maze air flow is unstable or diminished due to leaks in the tubing.

***Solution:*** Check for tears in the tubing attached to the T-maze, training/testing tubes, and odor delivery vials. Replace faulty tubing.

***Problem (Steps 51 and 52):*** T-maze air flow is unstable or diminished due to air holes in the T-maze elevator or in the training/testing tubes being blocked.

***Solution:*** Disassemble the maze and tubes and clean the obstructed holes.

***Problem (Steps 51 and 52):*** T-maze air flow is unstable or diminished due to the vacuum source being faulty.

***Solution:*** Check for faults in the vacuum source.

***Problem (Steps 51 and 52):*** T-maze air flow is unstable or diminished due to vacuum filters (Millipore) being blocked.

***Solution:*** Clean or replace Millipore filters.

***Problem (Steps 51 and 52):*** T-maze air flow is unstable or diminished due to T-maze clamps being loose.

***Solution:*** Replace the springs on the clamps, or switch to commercially available clamps (e.g., Quick-Grip Irwin 53006 Micro Clamps, black, 100 mm).

***Problem (Steps 64–68 and 91–94):*** T-maze does not move smoothly due to clamps or screws in the baseplate being too tight.

***Solution:*** Loosen the clamps on the T-maze and the screws in the baseplate. Hold the elevator in the middle and tighten the screws/clamps back. If necessary, use odorless silicone grease for moving parts.

***Problem (Steps 70, 71, 97, and 98):*** There are too few flies (total of less than 70) in the collection tubes after testing, due to flies being trapped in the vacuum port on the T-maze elevator.

***Solution:*** Sometimes, many flies get stuck in the middle portion of the elevator after testing, preventing them from being collected in any of the testing arms. This can happen due to excessive airflow from a few holes, which sucks the flies in. Ensure that the airflow is within a normal range and that the air holes in the testing port are unobstructed.

***Problem (Steps 70, 71, 97, and 98):*** There are too few flies (total of less than 70) in the collection tubes after testing, due to low number of surviving flies (can happen in appetitive conditioning experiments).

***Solution:*** You may notice dead flies in the agar vials following the starvation phase of the appetitive conditioning protocol. Older flies have decreased starvation-resistance, so use flies that are not older than 2–5 d after eclosion. To increase the survivability of flies when testing 24-h appetitive memory, feed the flies for 30 min after training and then transfer them back into food deprivation vials.

***Problem (Steps 70, 71, 97, and 98):*** There are too few flies (total of less than 70) in the collection tubes after testing, due to too few flies being aliquoted initially.

***Solution:*** When preparing flies for the next day’s experiments, make sure to aliquot 100 or more flies per vial.

***Problem (Steps 71 and 74):*** Low memory performance after aversive conditioning is observed due to debris in the shock grids.

***Solution:*** Gently scrub shock grids with appropriate material (e.g., abrasive paper P10000). Make sure not to damage the grid.

***Problem (Steps 71 and 74):*** Low memory performance after aversive conditioning is observed due to low humidity in the behavior room air.

***Solution:*** When performing aversive “shock” conditioning experiments, the humidity should be no lower than 55%. Check the humidity levels inside the behavior room.

***Problem (Steps 71, 74, 98, and 101):*** Low or unusually high memory performance is observed in control genotypes.

***Solution:*** Learning and memory–directed behavior is sensitive to changes in genetic background.

Different wild-type or control genotype flies may show variable levels of memory performance. Test setup using *Canton-S* flies (or other strain known to show normal performance) to distinguish between technical and biological problems.

***Problem (Steps 98 and 101):*** Low memory performance after appetitive conditioning is observed due to low-quality sugar paper.

***Solution:*** Occasionally, the sugar training may be less effective. This can occur if the sugar paper is not fully saturated with sugar. Ensure that a film of sucrose is present on top of the paper when preparing the sugar paper (see Fig. 15). Contamination in the sugar solution can also reduce the quality of the sugar paper; if this happens, prepare a new sucrose solution. Last, issues with sugar training can arise if the sugar paper is not completely dry. To ensure thorough drying, prepare the sugar paper several days in advance or dry it in a 31°C incubator.

***Problem (Steps 112, 117, and 120):*** T-maze airflow becomes unstable or diminished when heatsinks are used.

***Solution:*** Adjust the airflow while heatsinks are placed on the tubes.

***Problem (Steps 115 and 120):*** Fly distribution is abnormally skewed during testing due to an underlying, naive, color bias, independent of learned preference.

***Solution:*** LED intensity may not be ideal. Change the intensity until 50/50 distribution between colors is achieved. Lowering the intensity of either color light (green or blue) should reduce attraction to that color.

## Recipes

### Cornmeal Fly Media

**Reagents**

Agar (80–100 mesh; e.g., Fisher Bioreagents BP2641-1)

Autolyzed yeast (e.g., whole inactive yeast powder; Kerry Ingredients & Flavours 20050488)

D-glucose anhydrous (e.g., Fisher Chemical G/0500/70) Ethanol (100%)

Organic maize flour (e.g., gluten-free organic maize flour; Doves Farm Foods)

Reverse osmosis water

Tegosept (methyl 4-hydroxybenzoate; e.g., Sigma-Aldrich H3647-1KG)

**Equipment**

Beaker Bottle, 1-L

Cling film

Cooking pots (two: one with capacity >1.5-L and with a lid, one with capacity >0.5-L)

Fly vials (e.g., 25-mm × 95-mm polystyrene *Drosophila* vials; Flystuff 32-109)

Food mixer (e.g., Brentwood Appliances BTWSM1162BK Five-Speed Stand Mixer)

Hot plate

Metal spoons for stirring (two, large)

Mixing bowl (capacity >1-L)

Muslin cloth Pipettes (25-mL)

Pipettor (battery operated)

Sieve (metal, large; e.g., John Lewis stainless steel sieve/mesh colander, 22.5-cm) Thermometer

### Method

1. Mix 27.5 g of yeast powder and 52 g of maize flour in a mixing bowl.
2. Add 170 mL of water to the mixture.
3. Blend with a food mixer (or by hand with a spoon) until mixture is thin and smooth with no or few lumps. The consistency should be thick yet viscous enough to be poured through a sieve. Add more water if the consistency is too thick.
4. To filter out any remaining lumps, pour the mixture through a sieve into the smaller cooking pot. Use the metal spoon to break up any lumps remaining on the sieve and to push them through.
5. Add 935 mL of water to the other, larger cooking pot.
6. Add 7.9 g of agar to the pot in Step 5, distributing evenly over the water, and mix.
7. Using the hot plates, heat the mixture to 90°C and check that the agar is dissolved. The solution should be transparent, with a golden color.
8. Add 110 g of anhydrous D-glucose to the molten agar and continue mixing.
9. Add the yeast/maize flour mixture from Step 4 into the pot with agar and D-glucose. Continue mixing.
10. Bring the temperature of the mixture to 94°C, which should sterilize it. Maintain the boil for a period of 5 min. Keep stirring to avoid burning the mixture and keep the lid on to prevent evaporation.
11. Cool mixture to 72°C.
12. Combine 2.4 g of Tegosept and 9.2 mL of ethanol in a small beaker and mix until the Tegosept has dissolved. Add the Tegosept solution into the mixture that is now at 72°C (Step 11). Keep stirring and ensure that the Tegosept is mixed in well.
13. Pour the mixture into the 1-L bottle and pipet into vials (~2.5 mL per vial) before the food solidifies.
14. Cover the vials/boxes of vials with muslin cloth and allow them to dry. Once dry, remove the muslin cloth, wrap the boxes of vials in plastic to avoid overdrying, and store for up to 1 mo at room temperature.
  *This recipe is for preparing 1 L of fly food, enough for about 400 (±20) vials at 2*.*5 mL per vial*.

## Figures and Tables

**Table 1:** Printed parts (for one fly collection device)

Description	File name^a^	Quantity	Material
Funnel	Funnel.STL	1	Clear
Collection cylinder	Cylinder.STL	1	Clear

a Files are available at https://github.com/CNCBWaddellTmaze/TMaze/releases/tag/TMaze-v1.0. Onefly collection device is sufficient for use with multiple mazes.

**Table 2:** Hardware parts (for one T-maze)

Brand/supplier	Description	Part no.	Quantity
RS	M5 × 60-mm socket screw (three for clamp and three for pipe mount)	304-4564	6
RS	M5 washer (three for clamp and three for pipe mount)	525-931	6
RS	M5 wingnut (clamp)	521-866	3
RS	Spring (clamp)	751-578	1^a^
RS	M6 × 25-mm socket screw (base)	483-8332	4
RS	M5 dome nut, nylon (pipe mount)	232-6975	3
RS	M5 nut (pipe mount)	525-903	3
Fisher Scientific	Masterflex 45501-66 female Luer × 1/4-in to 28 UNF thread (pipe mount and tube caps)	11760279	12
Fisher Scientific	Masterflex 45504-08 male Luer with lock ring × 3/16-in hose barb (pipe mount and tube caps)	15216088	12
RS	M6 × 16 screw (nylon) (center)	232-6981	1
Polymax	O-ring BS020 (nitrile) (center)	BS020N70	3
Polymax	O-ring BS019 (nitrile) (tubes)	BS019N70	9
Polymax	O-ring BS016 (nitrile) (pipe mount)	BS016N70	2
Polymax	O-ring BS022 (Teflon) (center)	BS022PT	4
RS	Thread seal tape (pipe mount)	183-3490	1
Nalgene/Thermo	50 platinum-cured silicone tubing	8060-0020	2-m
Scientilic			
Kartell	Quick disconnectors, PE 4-5-6-mm, tapered	12549746	15
RS	Molex precrimped leads (KK) (shock tube)	126-0325	2
RS	Molex female connector housing (KK) (shock tube)	679-5287	2
Custom-made	Shock grids *Aversive conditioning experiments will require custom-made shock grids (such as those etched on copper-coated polyimide film or conductive indium tin oxide [ITO] film, as described by Semelidou et al. 2019 and Villar et al. 2022). The color/tint of the film on which the grid is laid may affect the delivery of visual stimuli in experiments (e.g., optogenetics or multisensory learning).*	—	2
Falcon	Nonpyrogenic serological 1-mL glass pipette (odor delivery vial)	357,521	2
Fisherbrand	EPA screw-neck 30-mL glass vials (odor delivery vial)	10758874	2
Fisherbrand	24-mm PP screw cap and seal (odor delivery vial)	11778266	2
RS	4-mm banana plug cable^b^ (one red and one black)	884-8926	2
RS	Molex two-way straight pin header^b^	483-8461	2

a Three segments 5- to 10-mm in length to be cut off the one larger spring.

b Custom cable for shock tubes is created by cutting banana cables in half and soldering to Molex two-way pin header. Quantities listed in the table above are based on conditioning experiments that use two odors.

**Table 3:** Hardware parts for switchbox

Brand/supplier	Description	Part no.	Quantity
RS	Panel-mount BNC connector	546-4910	1
RS	Toggle switch	734-7154	2
RS	4-mm red female banana socket	224-7423	2
RS	4-mm black female banana socket	224-7427	2
RS	Wire	267-5783	1-m
RS	Enclosure	918-8724	1

One switchbox is sufficient for use with two T-mazes.

**Table 4:** Printed parts (for one T-maze)

Description	File name^a^	Quantity	Material
Center part (elevator)	Centre.STL	1	Clear
Tubing adaptor mount	Centre Pipe Mount.STL	1	Clear
Right wall	Wall—RIGHT.STL	1	Clear
Left wall	Wall—LEFT.STL	1	Clear
Base	Base.STL	1	Any
Clamp	Clamp.STL	6	Clear
Tube	Fly tube V6.STL	7	Clear
Tube (for shock grid)	Fly tube V6—Shock.STL	2	Clear
Tube cap	Tube cap V3.STL	7	White
Tube cap (for shock grid)	Tube cap V3—Shock.STL	2	White
LED PCB cover	TMaze LED PCB—1.STL	4	White
	TMaze LED PCB—2.STL		

a Files are available at https://github.com/CNCBWaddellTmaze/TMaze/releases/tag/TMaze-v1.0. Quantities listed in the table above are based on conditioning experiments that use two odors.
